# Supplementary material for: Sum It Up for Me: A Novel Workshop in the Synthesis of Comprehensive Summary Statements for Pediatric Residents
Source: MedEdPORTAL. 2025 Nov 18;21:11555. doi: 10.15766/mep_2374-8265.11555 (PMC12623508; doi:10.15766/mep_2374-8265.11555)
Supplement: Supplementary file 1 — Participant Presurvey With Case.docxSum It Up For Me With Instructor Guide in Notes.pptxSmall-Group Cases for Instructors.docSmall-Group Cases for Learners.docParticipant Postsurvey With Case.docxSummary Statement Scoring Rubric.docx [file mep_2374-8265.11555-s001.zip › F. Summary Statement Scoring Rubric.docx]

| **Component** | **Scoring** | **Description** |
| --- | --- | --- |
| **Factual accuracy** | 0, 1 | Only accurate information included,  no misleading information  0 = No 1 = Yes |
| **Appropriate narrowing of differential diagnosis** | 0, 1, or 2 | Including key features to narrow the differential diagnosis;  0 = Does not narrow  1 = Some narrowing but missing elements  2 = Appropriately narrows |
| **Transformation of information** | 0, 1, or 2 | Use of medical terminology  0 = None 1 = Some  2 = Frequent and appropriate |
| **Use of semantic qualifiers** | 0, 1, or 2 | Use of qualitative terms, binary in nature  0 = None 1 = Some  2 = Frequent and appropriate |
| **Global rating** | 0, 1, or 2 | Overall rating of summary statement  0 = Significant problems/fatal flaws  1 = Adequate but can be improved  2 = Concise, complete, accurate |

*Adapted from "The Development and Preliminary Validation of a Rubric to Assess Medical Students’ Written Summary Statements in Virtual Patient Cases” by S. Smith, 2016, Acad Med Volume 91 (1), 94-100.*
