# Supplementary material for: Sum It Up for Me: A Novel Workshop in the Synthesis of Comprehensive Summary Statements for Pediatric Residents
Source: MedEdPORTAL. 2025 Nov 18;21:11555. doi: 10.15766/mep_2374-8265.11555 (PMC12623508; doi:10.15766/mep_2374-8265.11555)
Supplement: Supplementary file 1 — Participant Presurvey With Case.docxSum It Up For Me With Instructor Guide in Notes.pptxSmall-Group Cases for Instructors.docSmall-Group Cases for Learners.docParticipant Postsurvey With Case.docxSummary Statement Scoring Rubric.docx [file mep_2374-8265.11555-s001.zip › A. Participant Presurvey With Case.docx]

| Pre-Survey  **For the purposes of this survey, summary statements are defined as a one-sentence summary that highlights the defining features of a case. This can also be referred to as a one-liner or problem representation.** Describe your confidence with developing summary statements 1  2  3  4  5   \| Not Confident at all \| Extremely Confident \| \| --- \| --- \|  Describe your confidence with assessing the quality of summary statements 1  2  3  4  5   \| Not Confident at all \| Extremely Confident \| \| --- \| --- \|  An accurate summary statement should include the following (select the correct answer) Semantic Qualifiers  Pertinent Positives  Pertinent Negatives  Pertinent Positives and Pertinent Negatives  All of the above Select your training level  \| Medical Student  PGY-1  Other \|  \| \| --- \| --- \|  Are you a UPMC trainee or a visiting resident? UPMC trainee Visiting resident N/A. I am a medical student What is your residency training program?  \| Categorical Pediatrics  Combined Resident (Med-Peds, Peds Anesthesia, Triple board)  Other (Family Medicine) \|  \| \| --- \| --- \|  Review the provided case.In the space provided on the back of this sheet, write a summary statement based on this case | |  | | |
| --- | --- | --- | --- | --- | --- | --- | --- | --- | --- | --- | --- | --- |
| History of Present Illness:A 22-day-old female is presenting to the emergency room with vomiting and fever. The infant was well until six days prior to presentation, when she developed recurrent non-bloody, non-bilious emesis. Despite the vomiting, the infant appeared well and was behaving normally. On the day of presentation, she had a fever at home to 101.4℉, and so parents brought the infant to the emergency room for evaluation. She did not have respiratory symptoms, nasal congestion, rash, or diarrhea. Parents were concerned that she had intermittent, left-sided upper and lower extremity jerking movements while awake that lasted for less than 1 minute. She continued to breastfeed for about 15 minutes per breast every 2-3 hours and made 8+ wet diapers a day. Mom reported normal stooling, about once a day. The family shared that several family members had also developed fever and congestion in the past week.Past Medical History: NoneBirth History:The infant was born via vaginal delivery at 40+6 weeks to a 32-year-old G4P4 without any past medical history. Mom was not on any medications other than prenatal vitamins during this pregnancy. The infant did not require any resuscitation after birth, did not require a NICU stay, and was discharged on day of life 2 from the hospital. Mom’s prenatal labs: O positive, antibody negative, Group B strep negative, HIV non-reactive, RPR non-reactive, Gonorrhea and Chlamydia unknown, HSV unknown, Hepatitis B immune.Past Surgical History: Frenotomy in first week of lifeFamily History: Non-contributoryAllergies: NoneMedications: Vitamin D 400 units dailyImmunization History: UnimmunizedSocial History: The infant lives at home with her mother, father, and older siblings. She is not in daycare. The family denies passive tobacco exposure.Vitals: Temperature 38.5℃ \| Heart Rate 165 beats per minute \| Blood Pressure 65/40 \| Respiratory Rate 34 breaths per minute \| SpO2 100% on room air \| Weight 4.84kgGeneral: Flushed and warm well-nourished, well-developed infant lying in cribHEENT: Normocephalic, atraumatic, anterior fontanelle open and flatNeck: Supple, however, difficult to assess full range of motion due to increased stiffness and increased fussinessCardiovascular: Tachycardic, regular rhythm, normal S1, S2, no murmursPulmonary: Lungs clear to auscultation bilaterally, no wheezes, crackles or rhonchiGastrointestinal: Abdomen soft, non-tender and non-distended. Her umbilical stump was almost completely healed without erythema or discharge. She had normal bowel sounds in all 4 quadrantsGenitourinary: Normal female external genitaliaNeurologic: She was alert, moving all extremities. The infant was fussy but consolable. Her tone was appropriate and she had normal Moro, palmar, plantar, and Babinski reflexes.Dermatologic: No rashes or skin lesionsLymph nodes: no significant cervical, axillary or inguinal lymphadenopathyDiagnostics:Complete Blood Count: WBC 20.1 with 27% neutrophils, 64% lymphocytes, 9% monocytes \| Hemoglobin 15 \| Platelets 399Basic Metabolic Panel: Sodium 138 \| Potassium 5.6 \| Chloride 105 \| CO2 23 \| BUN 14 \| Creatinine 0.30 \| Glucose 101Lumbar Puncture: 192,000 RBCs/mm3 \| 1341 WBCs/mm3\| Protein 207 mg/dL \| Glucose 33 mg/dLChest X-ray: The cardiac silhouette is within normal limits, no focal consolidation. No vascular congestion, pleural effusion or pneumothorax.Urinalysis: Clear yellow urine, pH: 7.0, small leukocyte esterase, negative nitrites, negative glucose, negative bilirubin, specific gravity 1.005, No RBCs or WBCs, bacteria or squamous cellsRespiratory Viral Panel: Negative for Influenza A and B, Respiratory Syncytial virus, and Covid-19Pending studies: Urine culture, blood culture, Brain MRI, EEG | |  |  |  |
|  | |  |  |  |
|  | |  |  |  |
|  | |  |  |  |
|  | |  |  |  |
|  | |  |  |  |
|  | |  |  |  |
|  | |  |  |  |
|  | |  |  |  |
|  | |  |  |  |
|  | |  |  |  |
|  | |  |  |  |
|  | |  |  |  |
|  | |  |  |  |
|  | |  |  |  |
|  | |  |  |  |
|  | |  |  |  |
|  | |  |  |  |
|  | |  |  |  |
|  | |  |  |  |
|  | |  |  |  |
|  | |  |  |  |
|  | |  |  |  |
|  | |  |  |  |
|  | |  |  |  |
| Pre-Survey | |  |  |  |
