# Supplementary material for: Sum It Up for Me: A Novel Workshop in the Synthesis of Comprehensive Summary Statements for Pediatric Residents
Source: MedEdPORTAL. 2025 Nov 18;21:11555. doi: 10.15766/mep_2374-8265.11555 (PMC12623508; doi:10.15766/mep_2374-8265.11555)
Supplement: Supplementary file 1 — Participant Presurvey With Case.docxSum It Up For Me With Instructor Guide in Notes.pptxSmall-Group Cases for Instructors.docSmall-Group Cases for Learners.docParticipant Postsurvey With Case.docxSummary Statement Scoring Rubric.docx [file mep_2374-8265.11555-s001.zip › B. Sum It Up For Me With Instructor Guide in Notes.pptx]

## Slide 1
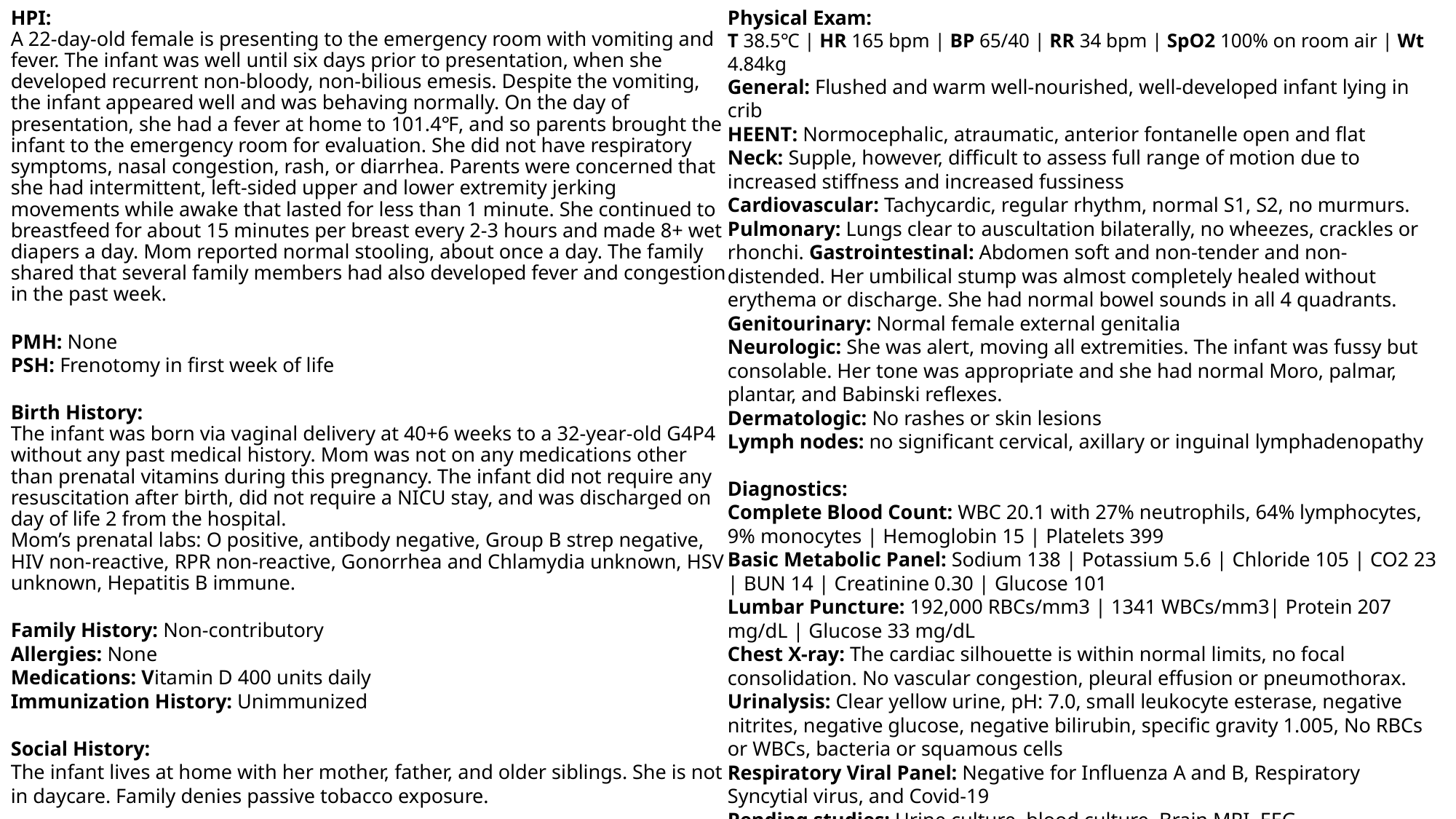

HPI:
A 22-day-old female is presenting to the emergency room with vomiting and fever. The infant was well until six days prior to presentation, when she developed recurrent non-bloody, non-bilious emesis. Despite the vomiting, the infant appeared well and was behaving normally. On the day of presentation, she had a fever at home to 101.4℉, and so parents brought the infant to the emergency room for evaluation. She did not have respiratory symptoms, nasal congestion, rash, or diarrhea. Parents were concerned that she had intermittent, left-sided upper and lower extremity jerking movements while awake that lasted for less than 1 minute. She continued to breastfeed for about 15 minutes per breast every 2-3 hours and made 8+ wet diapers a day. Mom reported normal stooling, about once a day. The family shared that several family members had also developed fever and congestion in the past week.
PMH: None
PSH: Frenotomy in first week of life
Birth History:
The infant was born via vaginal delivery at 40+6 weeks to a 32-year-old G4P4 without any past medical history. Mom was not on any medications other than prenatal vitamins during this pregnancy. The infant did not require any resuscitation after birth, did not require a NICU stay, and was discharged on day of life 2 from the hospital.
Mom’s prenatal labs: O positive, antibody negative, Group B strep negative, HIV non-reactive, RPR non-reactive, Gonorrhea and Chlamydia unknown, HSV unknown, Hepatitis B immune.
Family History: Non-contributory
Allergies: None
Medications: Vitamin D 400 units daily
Immunization History: Unimmunized
Social History:
The infant lives at home with her mother, father, and older siblings. She is not in daycare. Family denies passive tobacco exposure.
Physical Exam:
T 38.5℃ | HR 165 bpm | BP 65/40 | RR 34 bpm | SpO2 100% on room air | Wt 4.84kg
General: Flushed and warm well-nourished, well-developed infant lying in crib
HEENT: Normocephalic, atraumatic, anterior fontanelle open and flat
Neck: Supple, however, difficult to assess full range of motion due to increased stiffness and increased fussiness
Cardiovascular: Tachycardic, regular rhythm, normal S1, S2, no murmurs.
Pulmonary: Lungs clear to auscultation bilaterally, no wheezes, crackles or rhonchi. Gastrointestinal: Abdomen soft and non-tender and non-distended. Her umbilical stump was almost completely healed without erythema or discharge. She had normal bowel sounds in all 4 quadrants.
Genitourinary: Normal female external genitalia
Neurologic: She was alert, moving all extremities. The infant was fussy but consolable. Her tone was appropriate and she had normal Moro, palmar, plantar, and Babinski reflexes.
Dermatologic: No rashes or skin lesions
Lymph nodes: no significant cervical, axillary or inguinal lymphadenopathy
Diagnostics:
Complete Blood Count: WBC 20.1 with 27% neutrophils, 64% lymphocytes, 9% monocytes | Hemoglobin 15 | Platelets 399
Basic Metabolic Panel: Sodium 138 | Potassium 5.6 | Chloride 105 | CO2 23 | BUN 14 | Creatinine 0.30 | Glucose 101
Lumbar Puncture: 192,000 RBCs/mm3 | 1341 WBCs/mm3| Protein 207 mg/dL | Glucose 33 mg/dL
Chest X-ray: The cardiac silhouette is within normal limits, no focal consolidation. No vascular congestion, pleural effusion or pneumothorax.
Urinalysis: Clear yellow urine, pH: 7.0, small leukocyte esterase, negative nitrites, negative glucose, negative bilirubin, specific gravity 1.005, No RBCs or WBCs, bacteria or squamous cells
Respiratory Viral Panel: Negative for Influenza A and B, Respiratory Syncytial virus, and Covid-19
Pending studies: Urine culture, blood culture, Brain MRI, EEG

## Slide 2
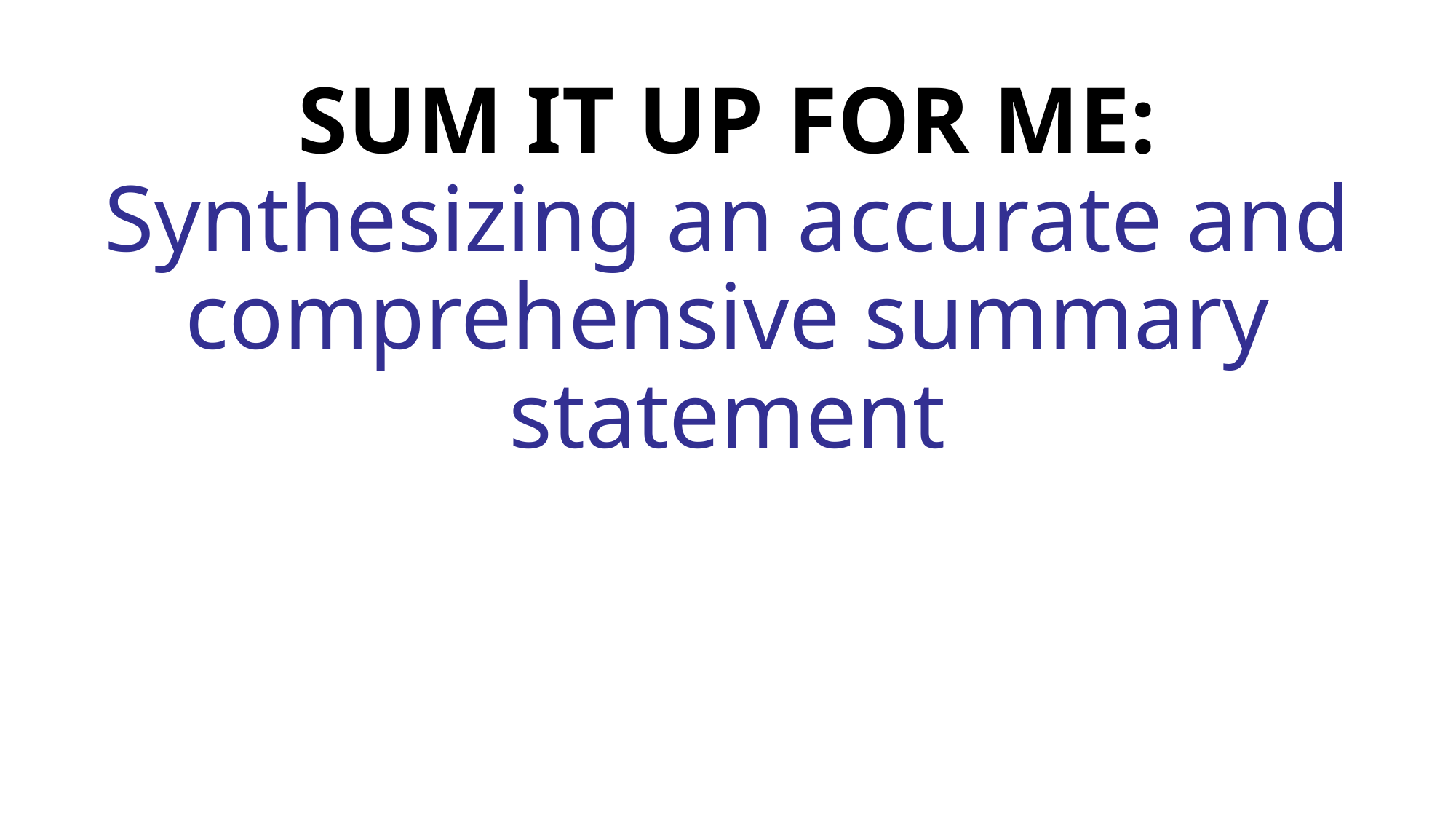

# SUM IT UP FOR ME: Synthesizing an accurate and comprehensive summary statement

## Slide 3
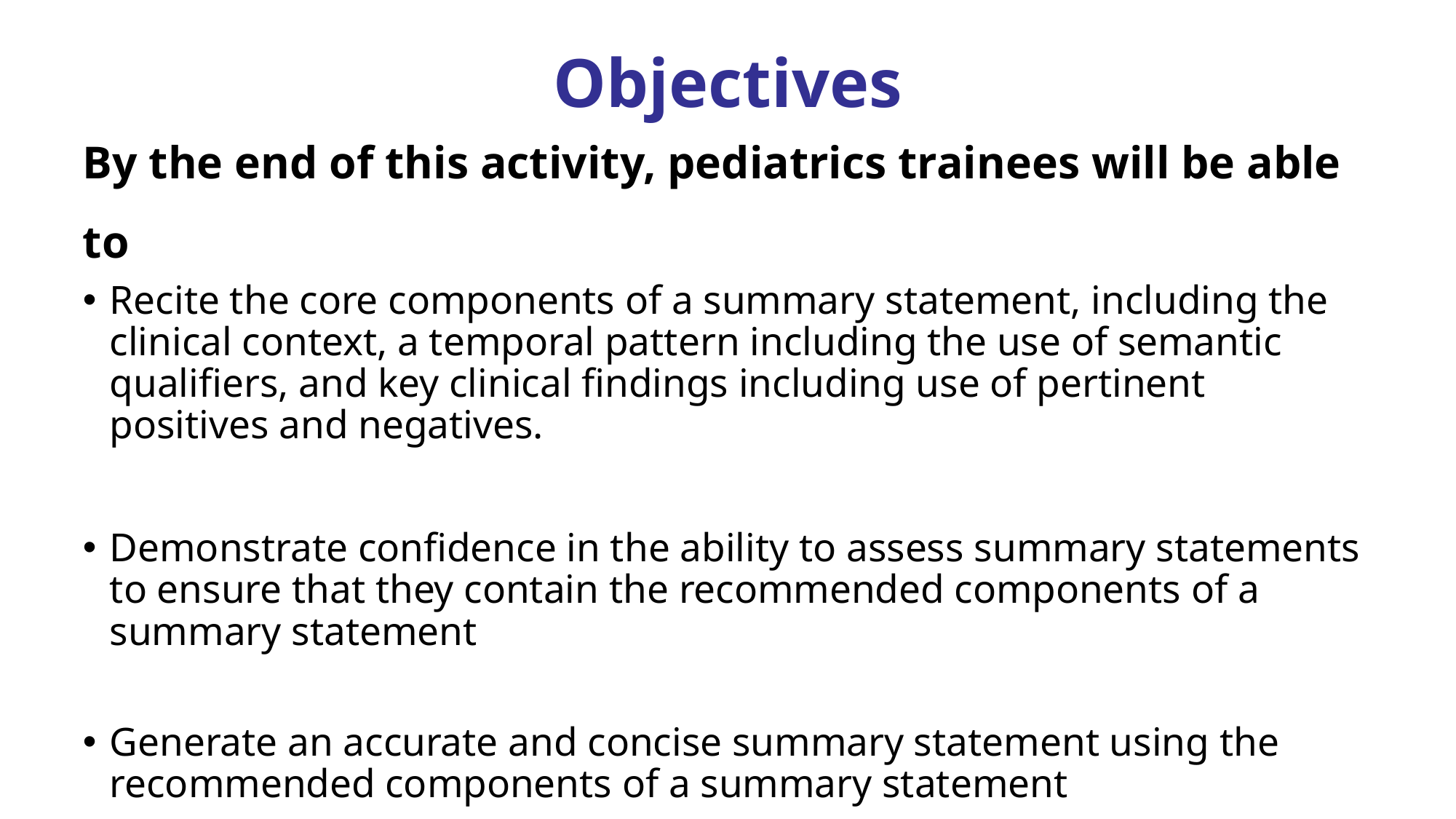

# Objectives
By the end of this activity, pediatrics trainees will be able to
Recite the core components of a summary statement, including the clinical context, a temporal pattern including the use of semantic qualifiers, and key clinical findings including use of pertinent positives and negatives.
Demonstrate confidence in the ability to assess summary statements to ensure that they contain the recommended components of a summary statement
Generate an accurate and concise summary statement using the recommended components of a summary statement

## Slide 4
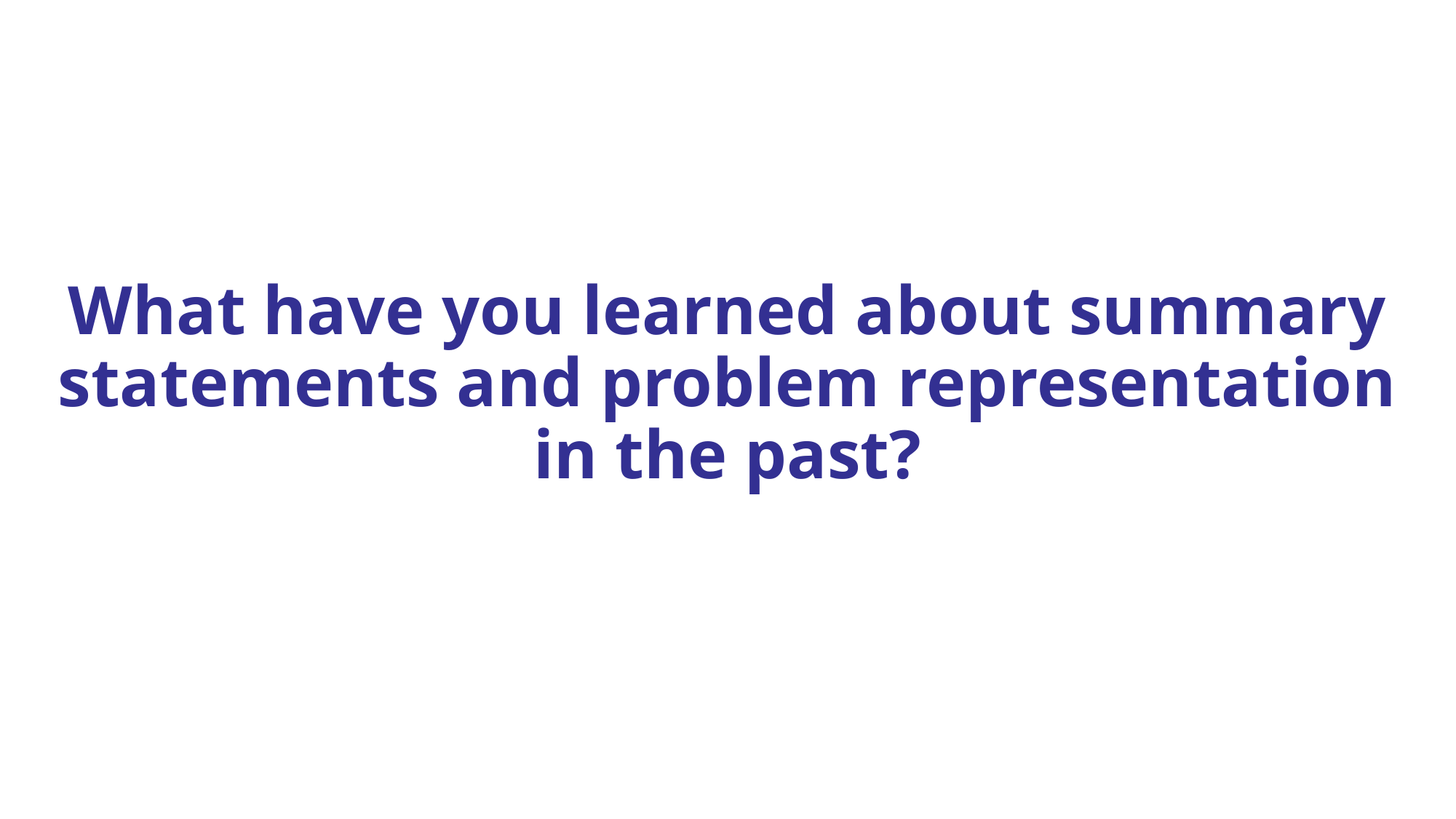

# What have you learned about summary statements and problem representation in the past?

## Slide 5
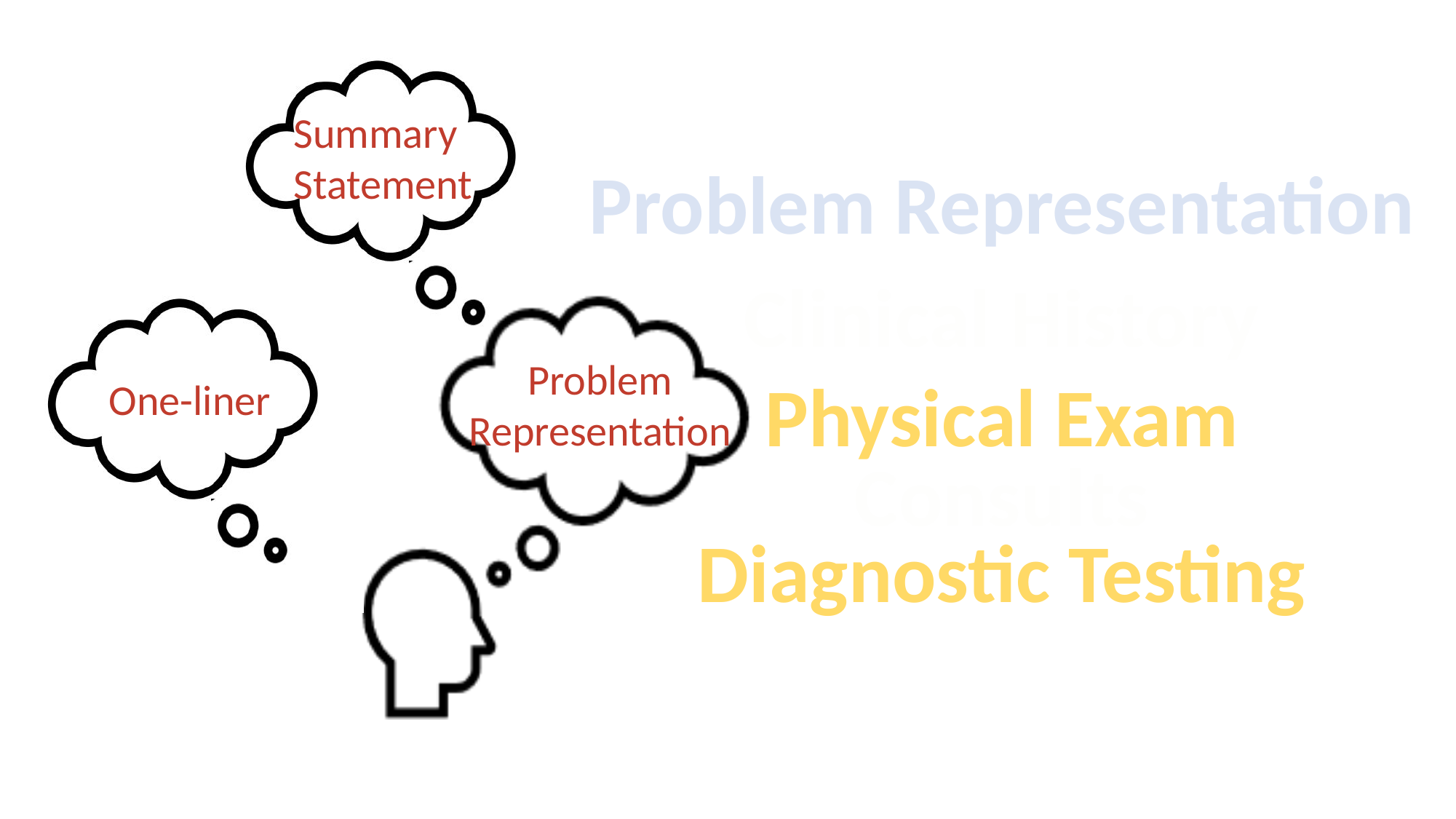

Summary Statement
Problem
Representation
One-liner
Problem Representation
Clinical History
Physical Exam
Consults
Diagnostic Testing

## Slide 6
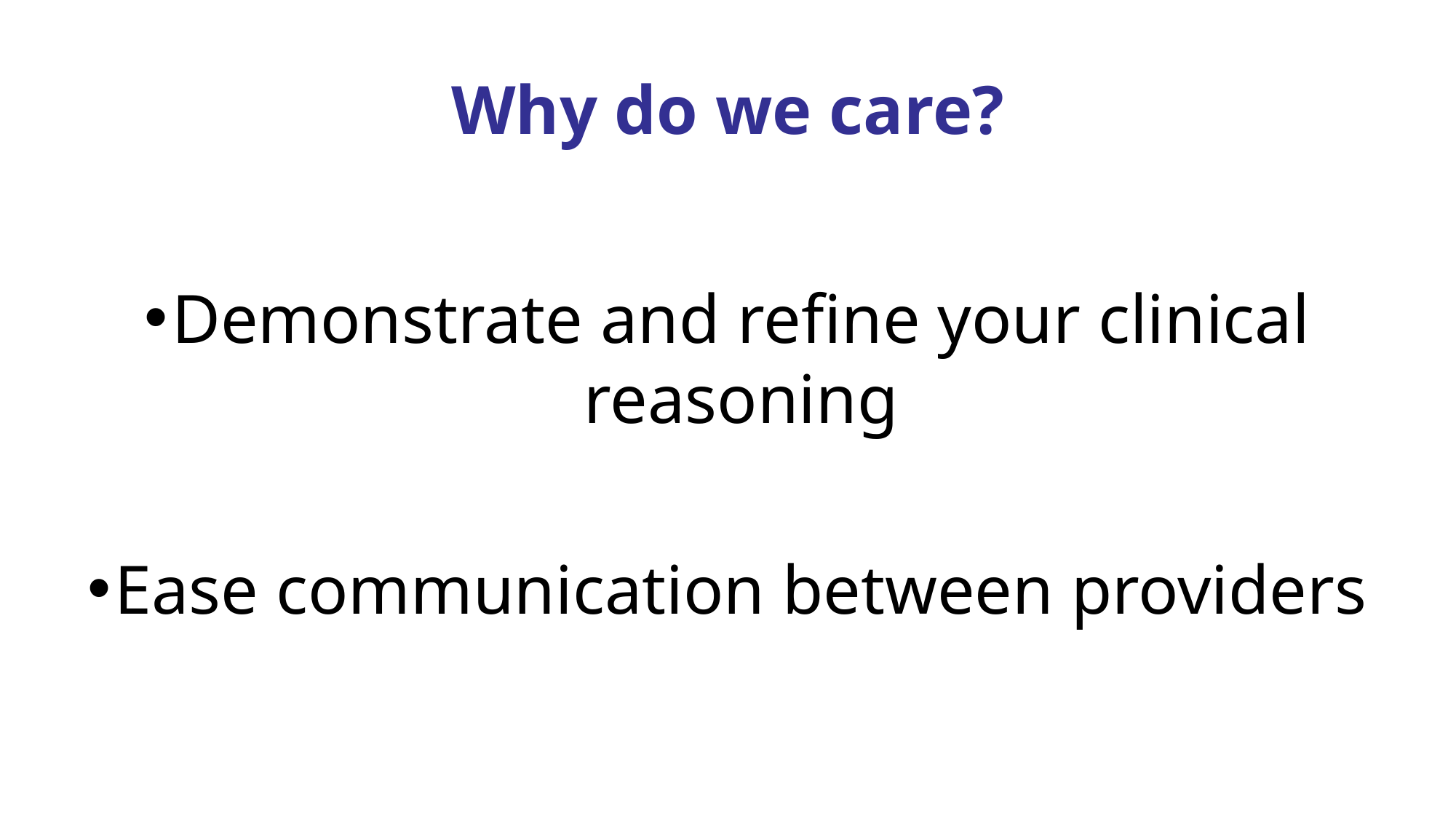

# Why do we care?
Demonstrate and refine your clinical reasoning
Ease communication between providers

## Slide 7
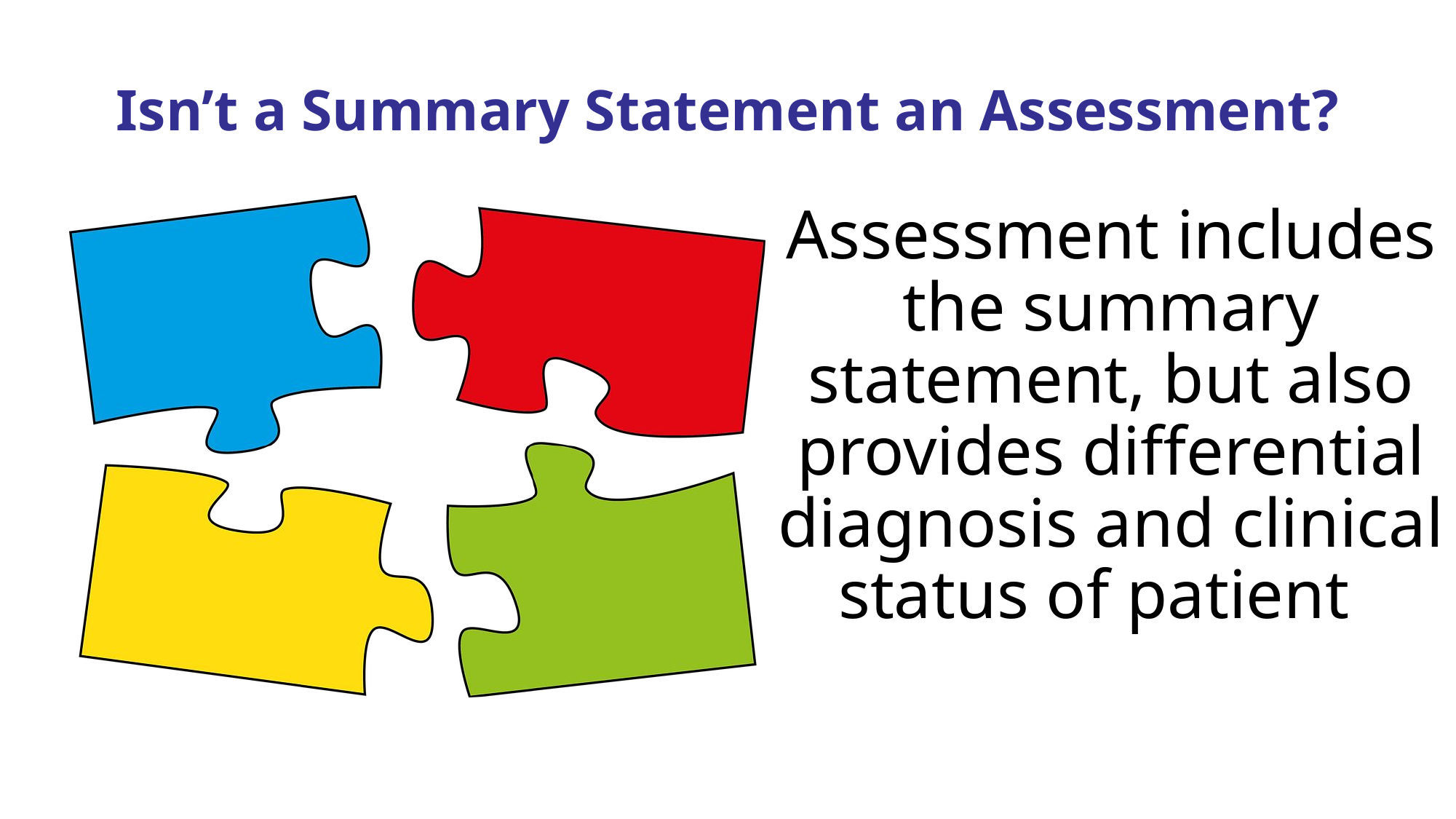

# Isn’t a Summary Statement an Assessment?
Assessment includes the summary statement, but also provides differential diagnosis and clinical status of patient

## Slide 8
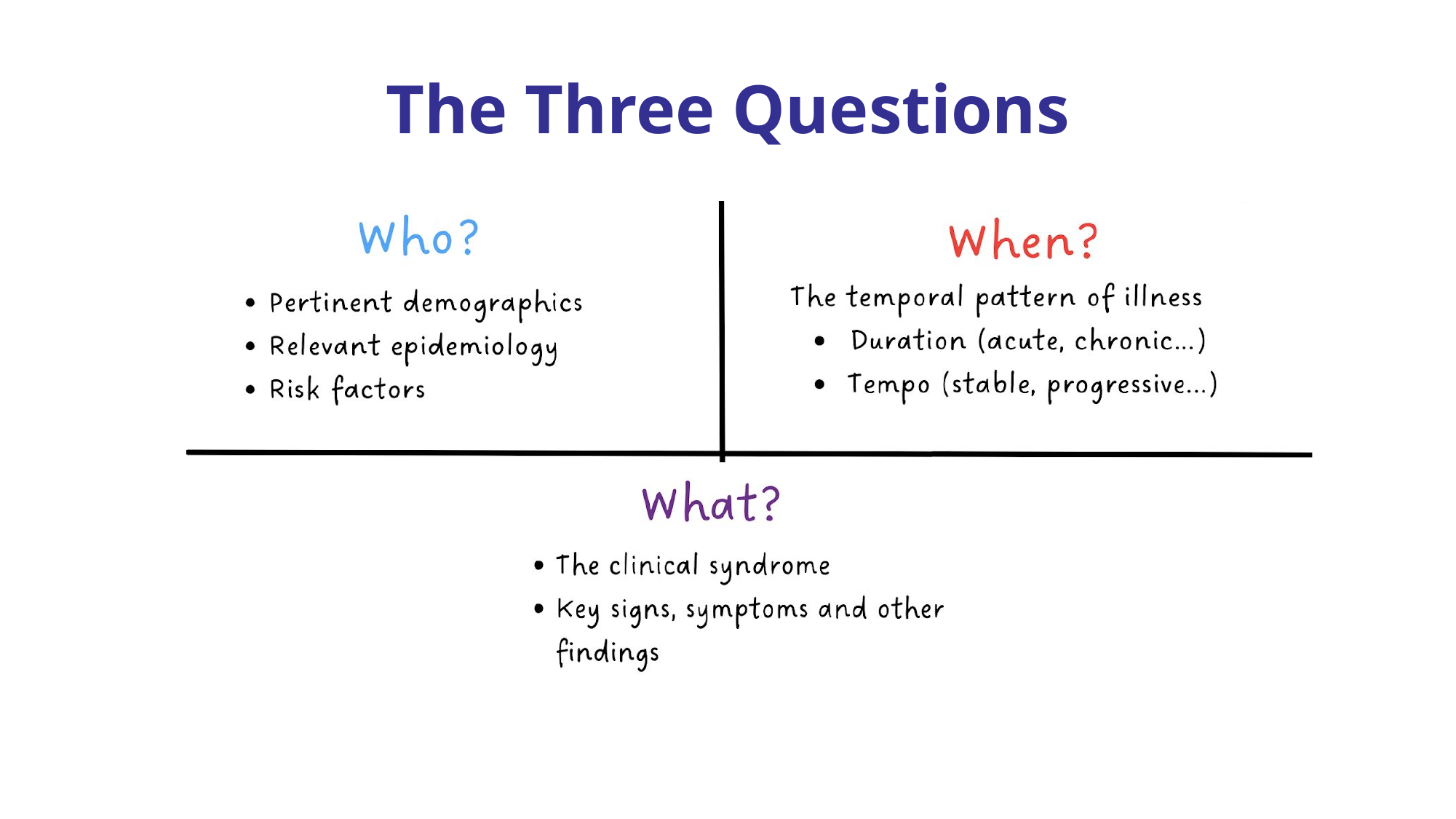

# The Three Questions

## Slide 9
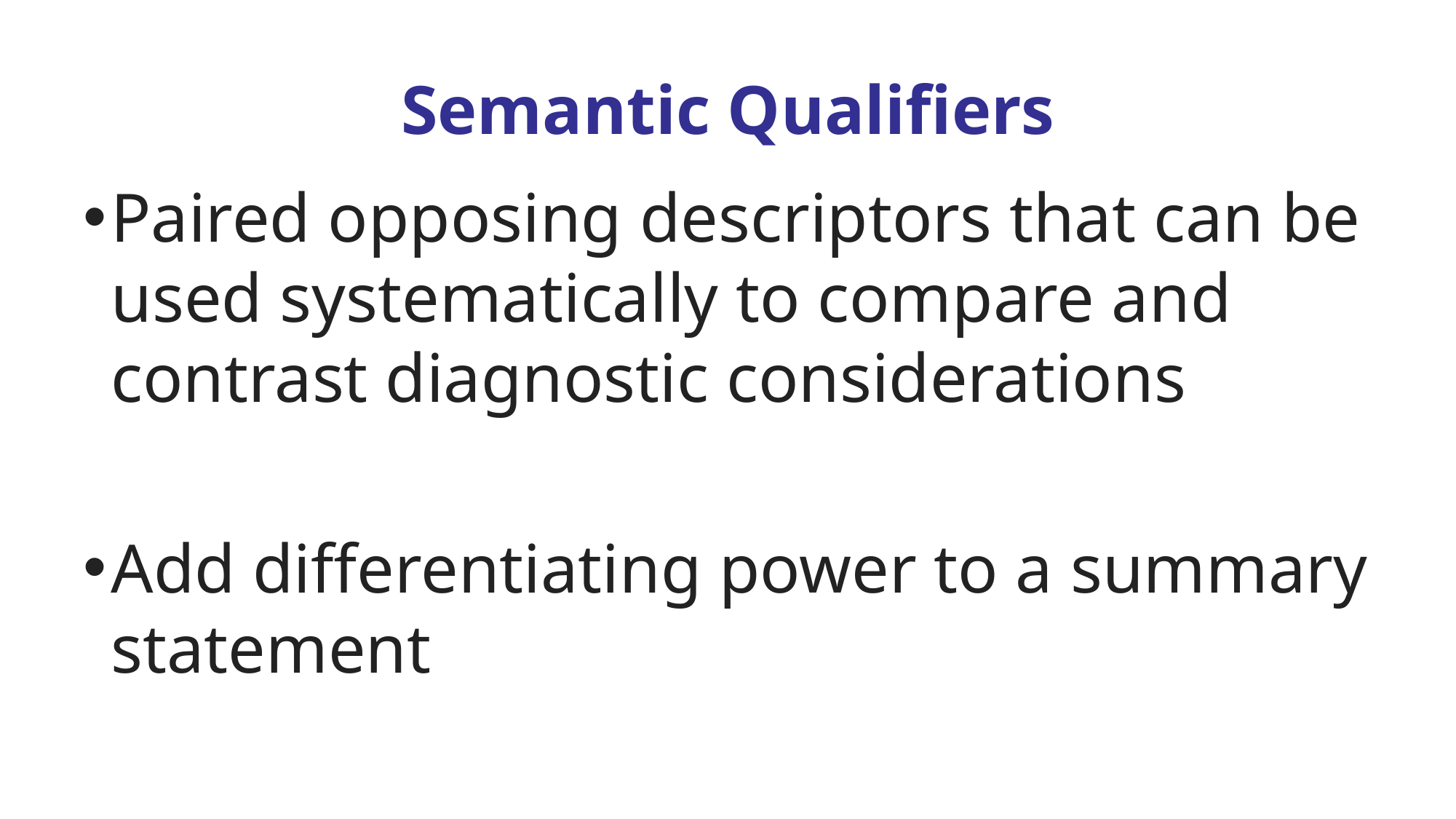

# Semantic Qualifiers
Paired opposing descriptors that can be used systematically to compare and contrast diagnostic considerations
Add differentiating power to a summary statement

## Slide 10
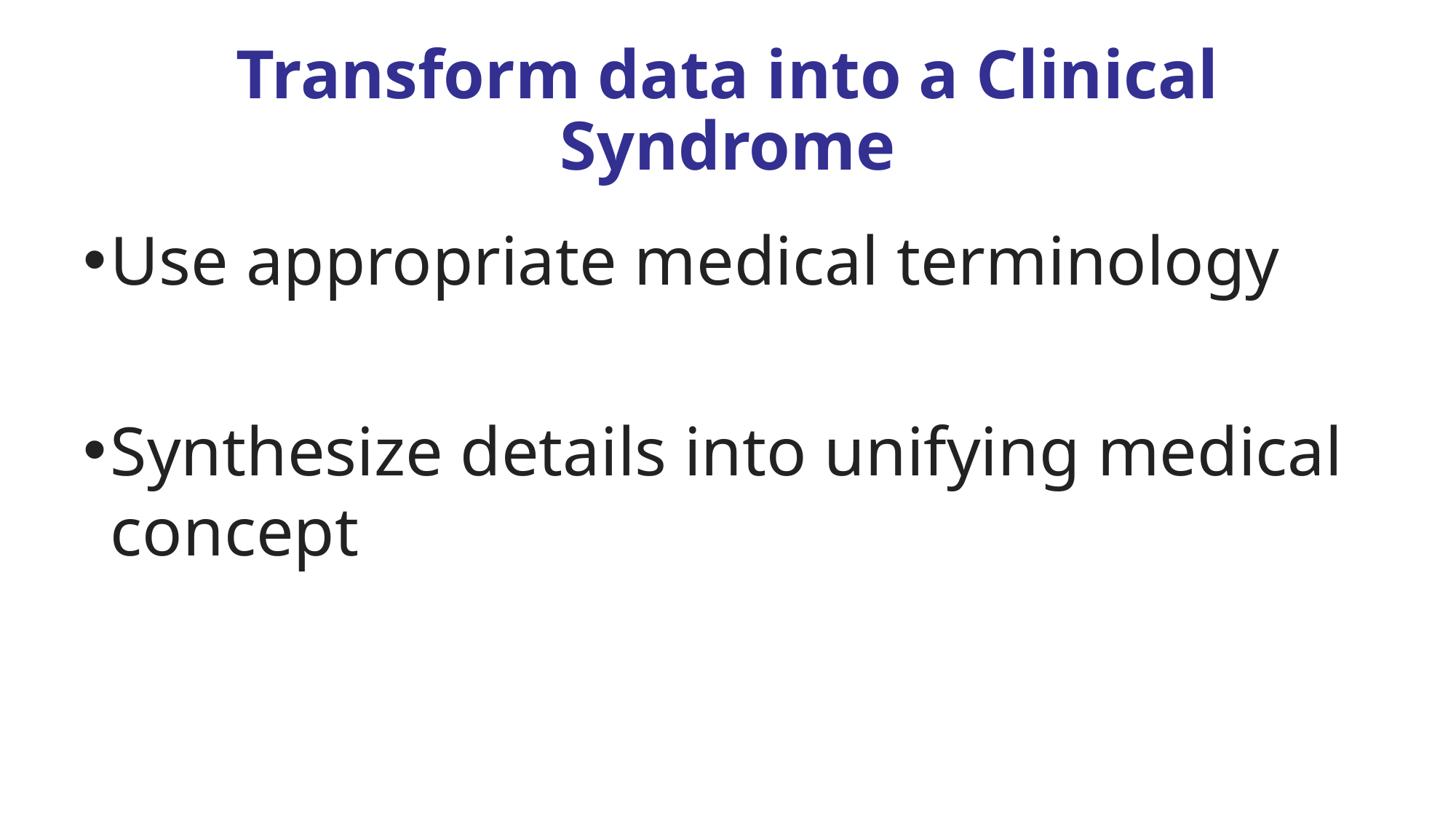

# Transform data into a Clinical Syndrome
Use appropriate medical terminology
Synthesize details into unifying medical concept

## Slide 11
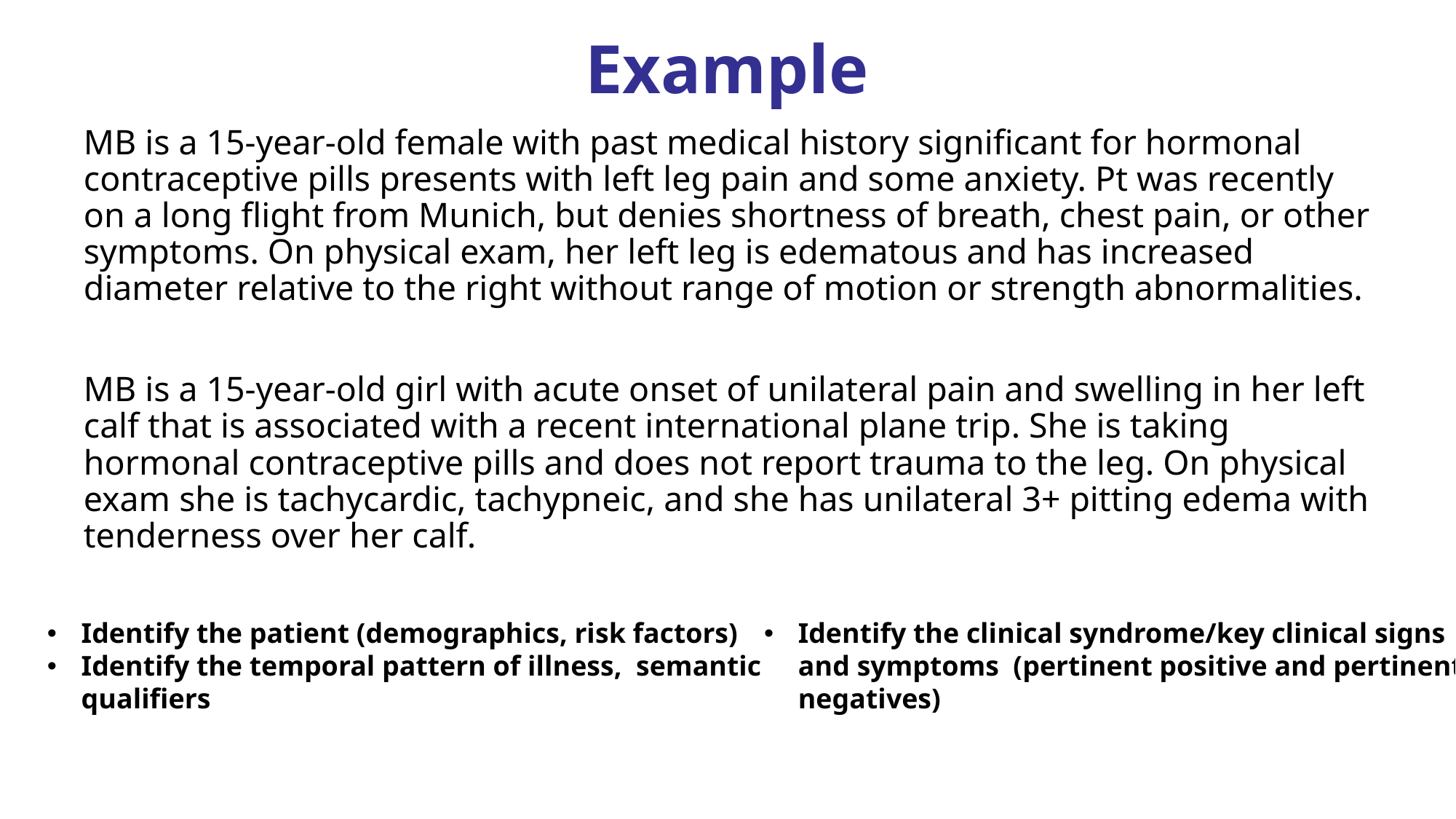

# Example
MB is a 15-year-old female with past medical history significant for hormonal contraceptive pills presents with left leg pain and some anxiety. Pt was recently on a long flight from Munich, but denies shortness of breath, chest pain, or other symptoms. On physical exam, her left leg is edematous and has increased diameter relative to the right without range of motion or strength abnormalities.
MB is a 15-year-old girl with acute onset of unilateral pain and swelling in her left calf that is associated with a recent international plane trip. She is taking hormonal contraceptive pills and does not report trauma to the leg. On physical exam she is tachycardic, tachypneic, and she has unilateral 3+ pitting edema with tenderness over her calf.
Identify the patient (demographics, risk factors)
Identify the temporal pattern of illness, semantic qualifiers
Identify the clinical syndrome/key clinical signs and symptoms (pertinent positive and pertinent negatives)

## Slide 12
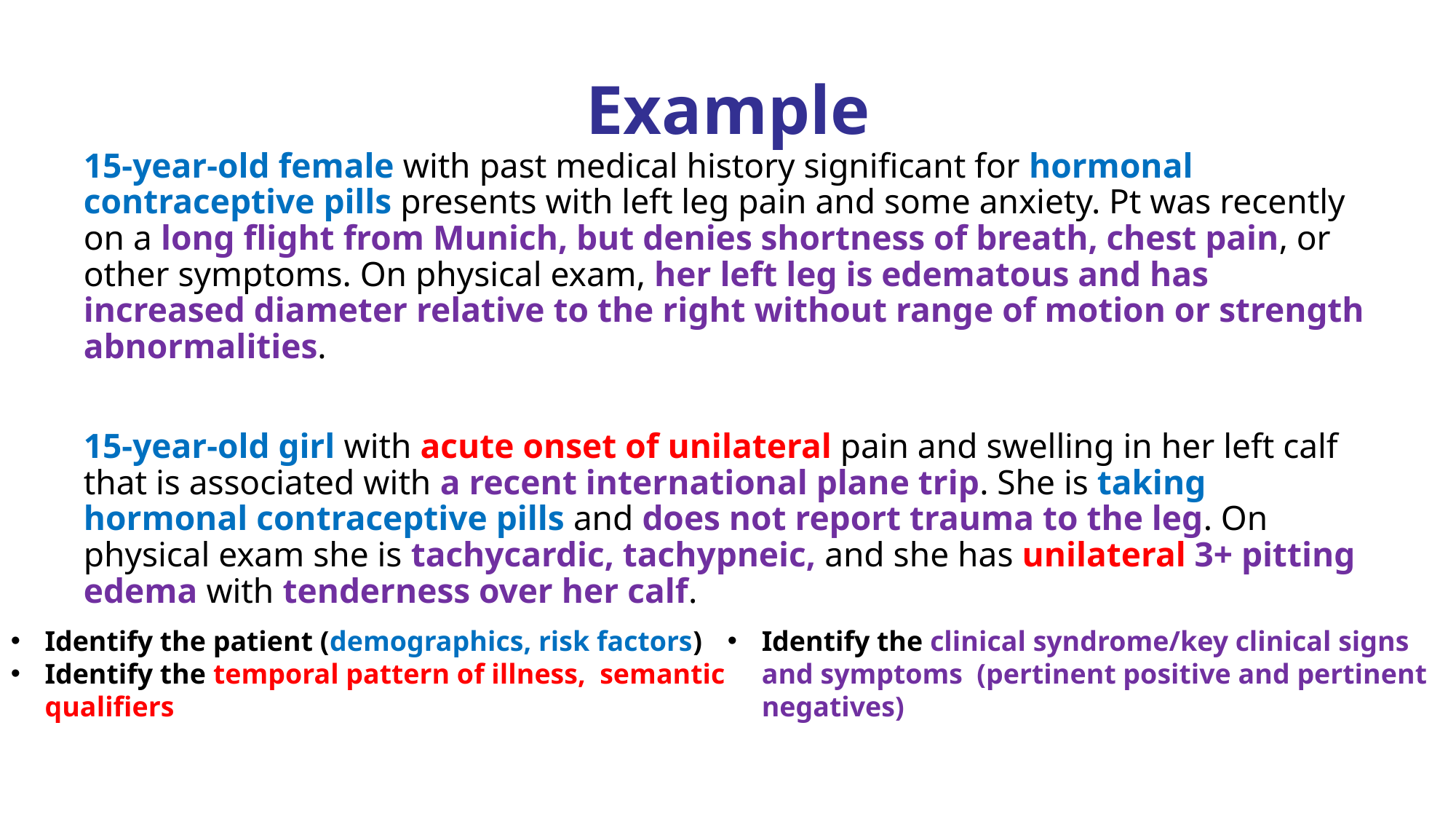

# Example
15-year-old female with past medical history significant for hormonal contraceptive pills presents with left leg pain and some anxiety. Pt was recently on a long flight from Munich, but denies shortness of breath, chest pain, or other symptoms. On physical exam, her left leg is edematous and has increased diameter relative to the right without range of motion or strength abnormalities.
15-year-old girl with acute onset of unilateral pain and swelling in her left calf that is associated with a recent international plane trip. She is taking hormonal contraceptive pills and does not report trauma to the leg. On physical exam she is tachycardic, tachypneic, and she has unilateral 3+ pitting edema with tenderness over her calf.
Identify the patient (demographics, risk factors)
Identify the temporal pattern of illness, semantic qualifiers
Identify the clinical syndrome/key clinical signs and symptoms (pertinent positive and pertinent negatives)

## Slide 13
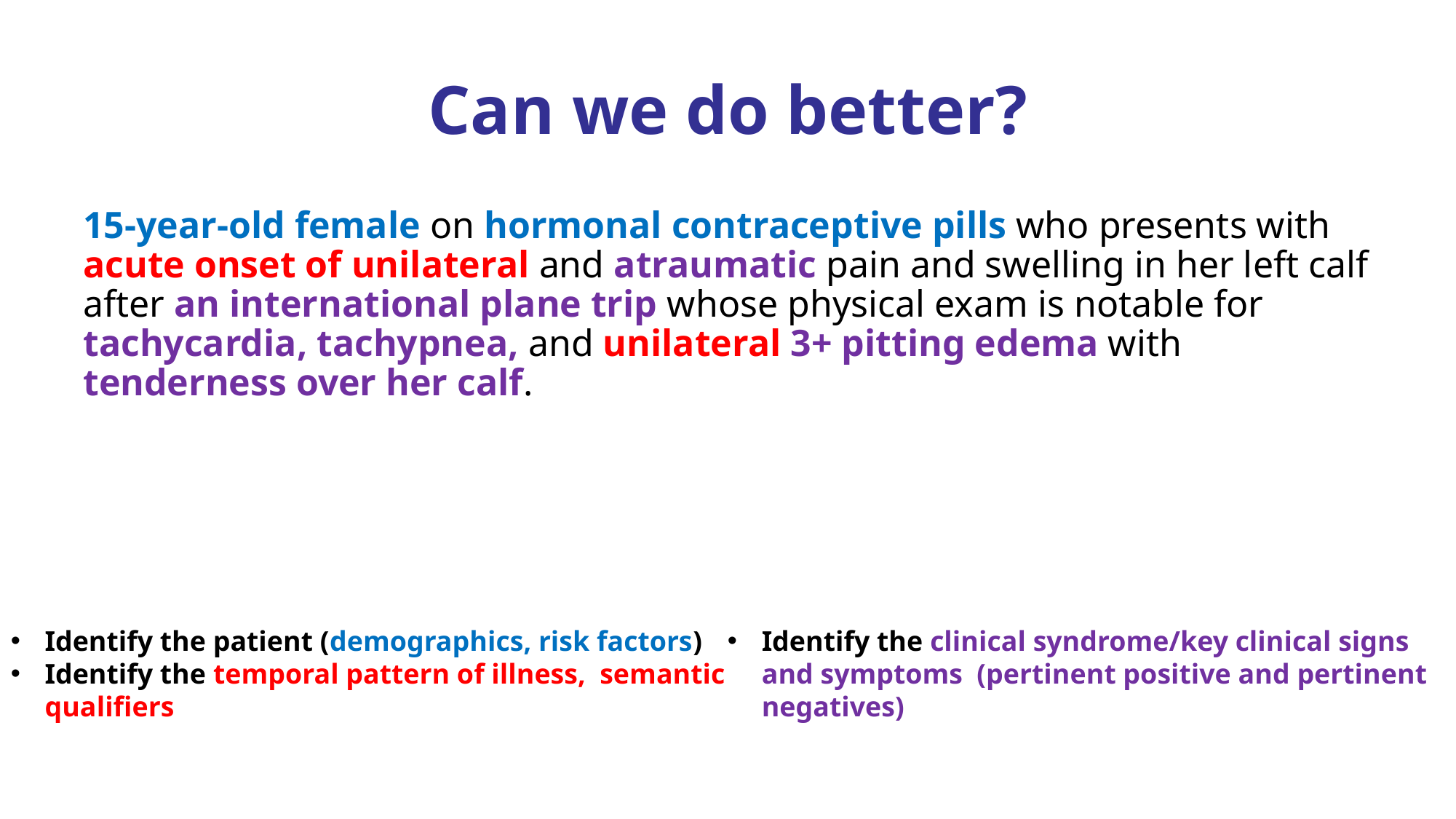

# Can we do better?
15-year-old female on hormonal contraceptive pills who presents with acute onset of unilateral and atraumatic pain and swelling in her left calf after an international plane trip whose physical exam is notable for tachycardia, tachypnea, and unilateral 3+ pitting edema with tenderness over her calf.
Identify the patient (demographics, risk factors)
Identify the temporal pattern of illness, semantic qualifiers
Identify the clinical syndrome/key clinical signs and symptoms (pertinent positive and pertinent negatives)

## Slide 14
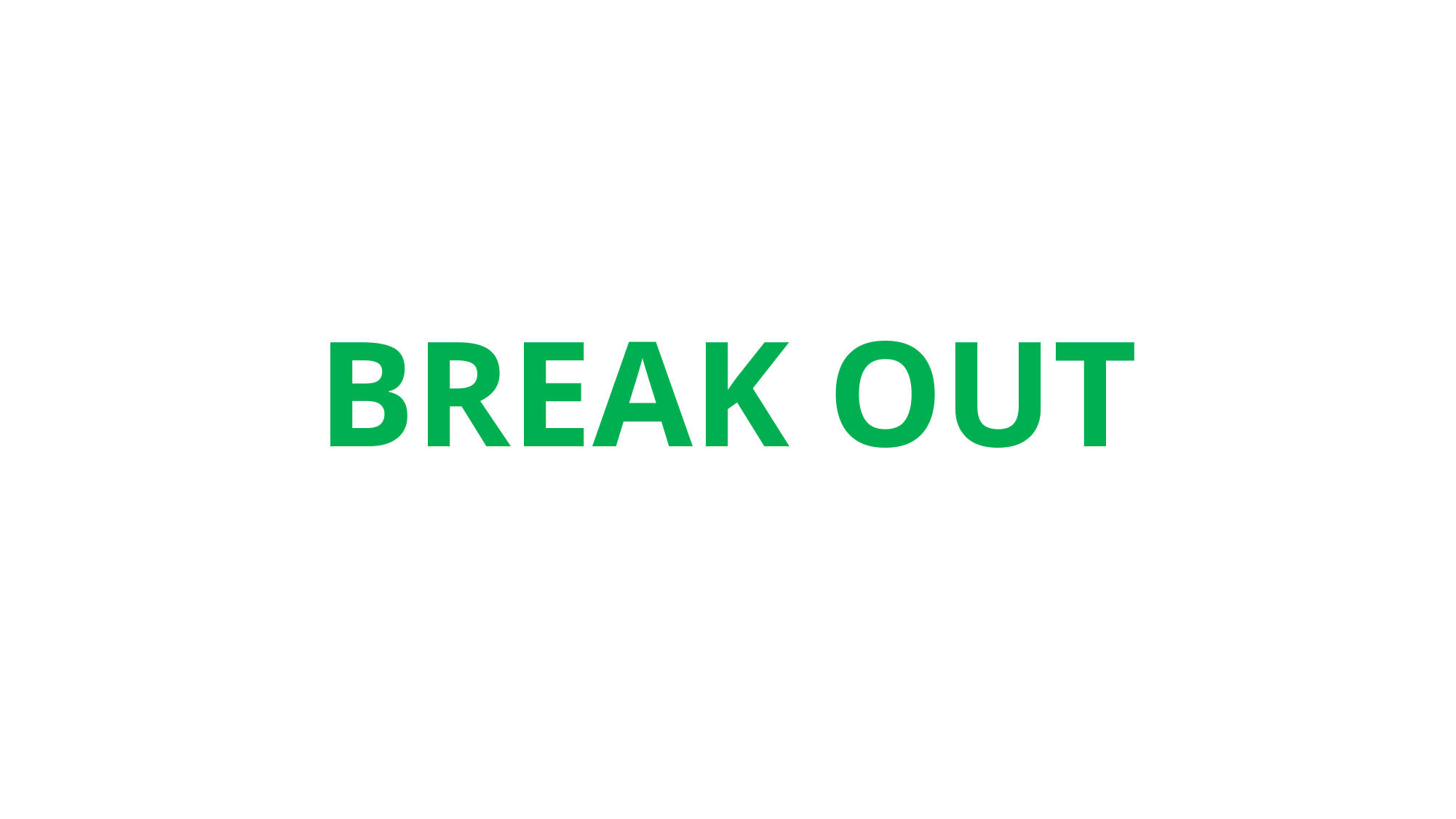

BREAK OUT

## Slide 15
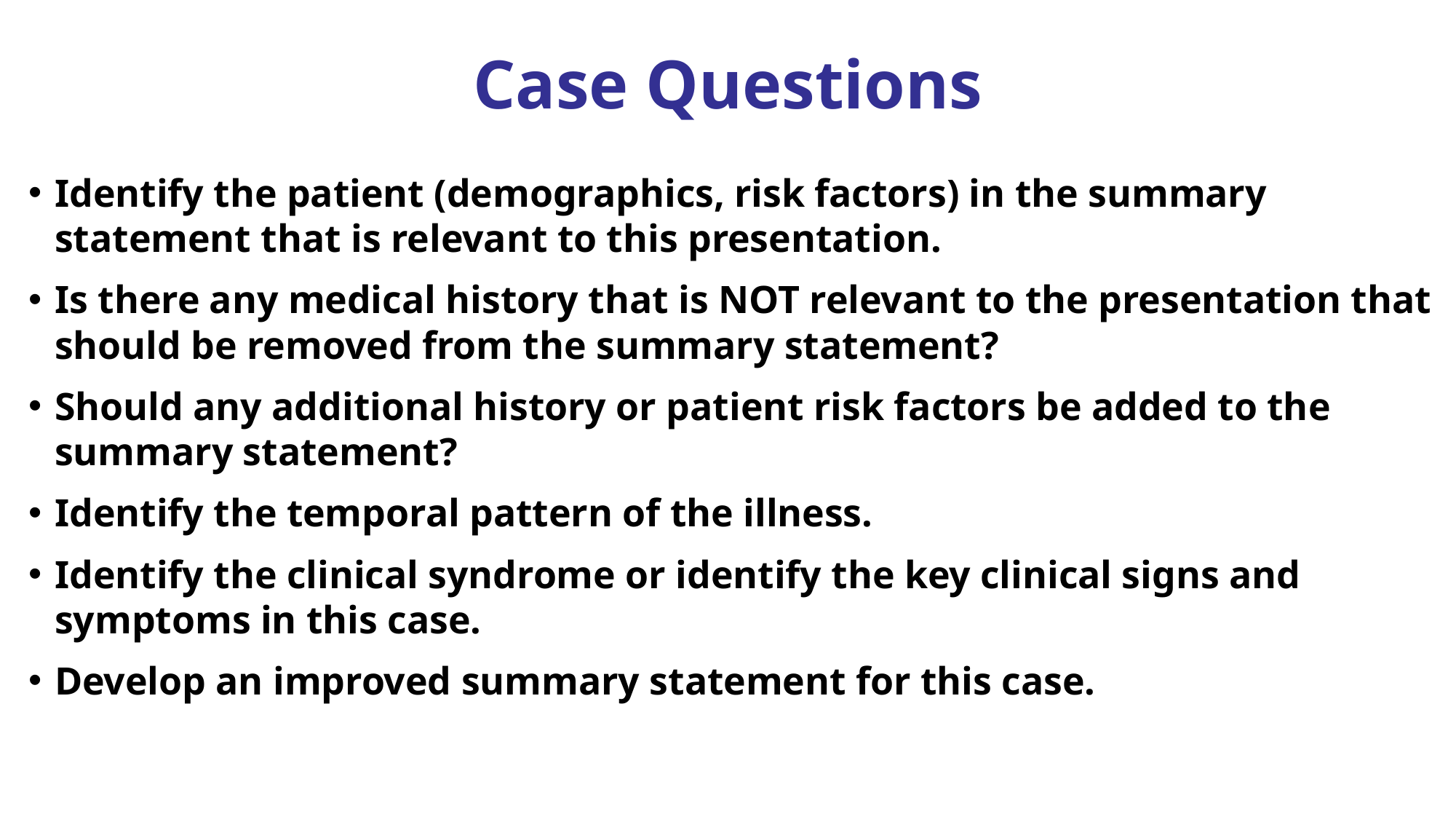

# Case Questions
Identify the patient (demographics, risk factors) in the summary statement that is relevant to this presentation.
Is there any medical history that is NOT relevant to the presentation that should be removed from the summary statement?
Should any additional history or patient risk factors be added to the summary statement?
Identify the temporal pattern of the illness.
Identify the clinical syndrome or identify the key clinical signs and symptoms in this case.
Develop an improved summary statement for this case.

## Slide 16
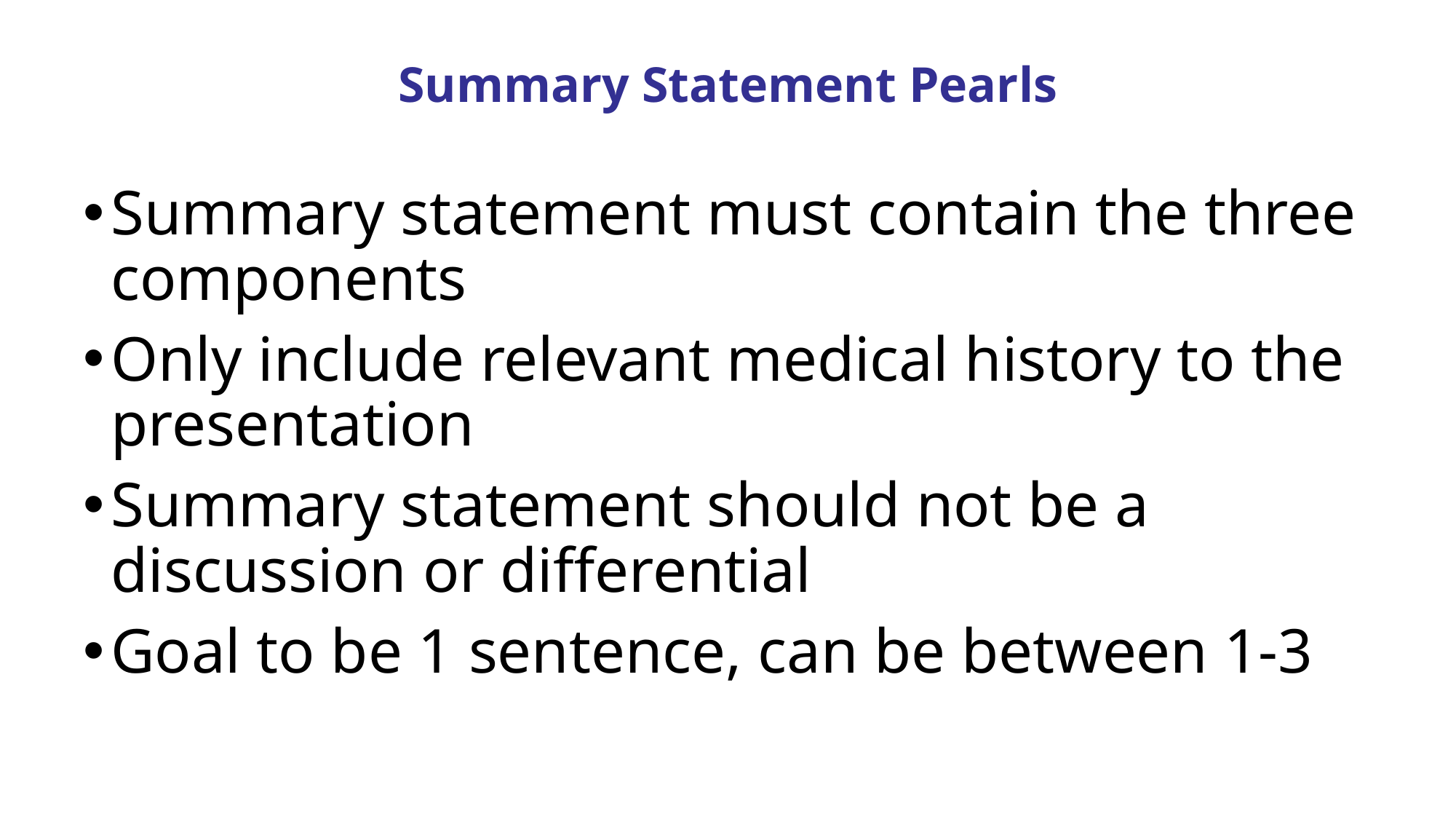

# Summary Statement Pearls
Summary statement must contain the three components
Only include relevant medical history to the presentation
Summary statement should not be a discussion or differential
Goal to be 1 sentence, can be between 1-3

## Slide 17
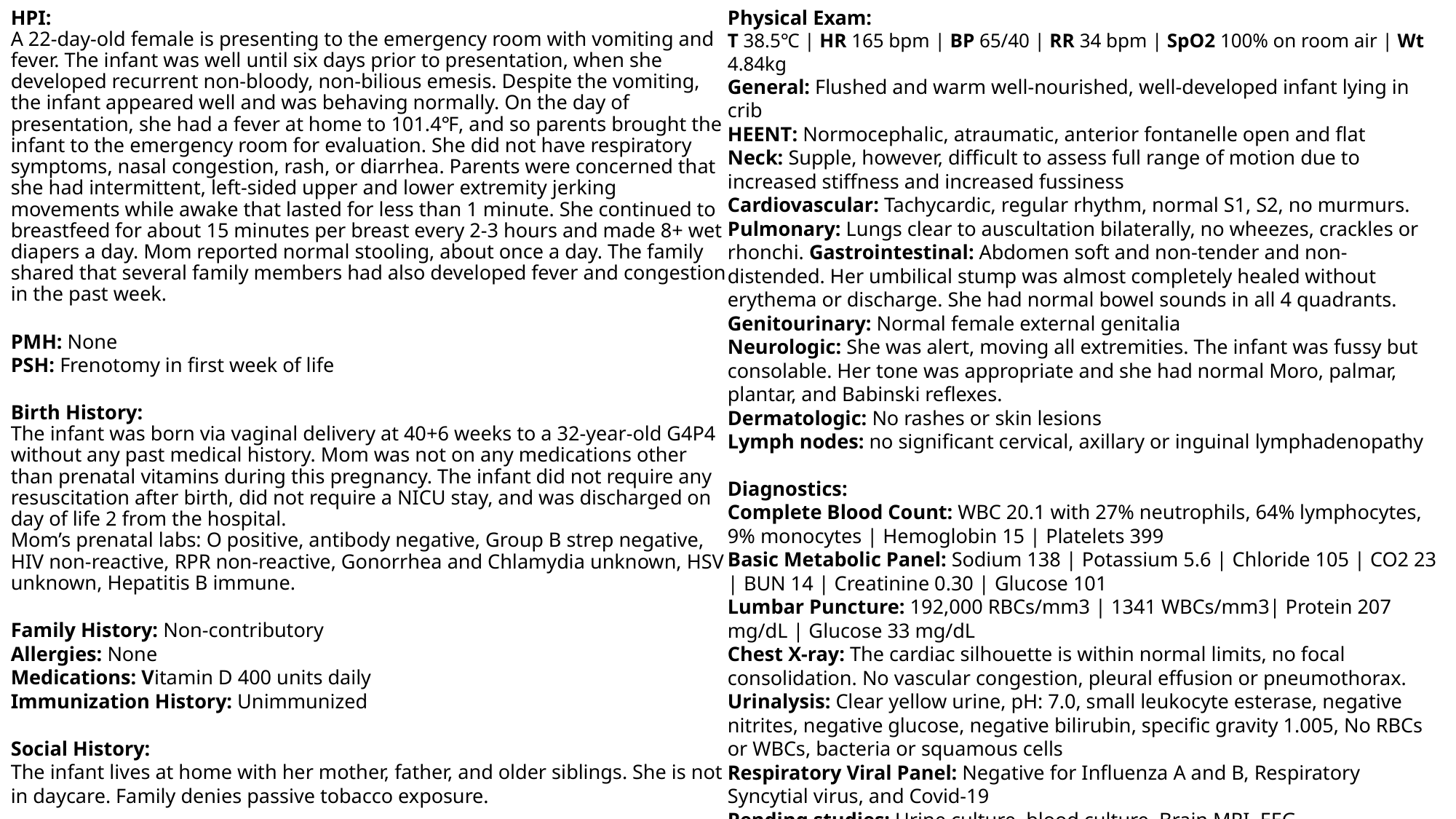

HPI:
A 22-day-old female is presenting to the emergency room with vomiting and fever. The infant was well until six days prior to presentation, when she developed recurrent non-bloody, non-bilious emesis. Despite the vomiting, the infant appeared well and was behaving normally. On the day of presentation, she had a fever at home to 101.4℉, and so parents brought the infant to the emergency room for evaluation. She did not have respiratory symptoms, nasal congestion, rash, or diarrhea. Parents were concerned that she had intermittent, left-sided upper and lower extremity jerking movements while awake that lasted for less than 1 minute. She continued to breastfeed for about 15 minutes per breast every 2-3 hours and made 8+ wet diapers a day. Mom reported normal stooling, about once a day. The family shared that several family members had also developed fever and congestion in the past week.
PMH: None
PSH: Frenotomy in first week of life
Birth History:
The infant was born via vaginal delivery at 40+6 weeks to a 32-year-old G4P4 without any past medical history. Mom was not on any medications other than prenatal vitamins during this pregnancy. The infant did not require any resuscitation after birth, did not require a NICU stay, and was discharged on day of life 2 from the hospital.
Mom’s prenatal labs: O positive, antibody negative, Group B strep negative, HIV non-reactive, RPR non-reactive, Gonorrhea and Chlamydia unknown, HSV unknown, Hepatitis B immune.
Family History: Non-contributory
Allergies: None
Medications: Vitamin D 400 units daily
Immunization History: Unimmunized
Social History:
The infant lives at home with her mother, father, and older siblings. She is not in daycare. Family denies passive tobacco exposure.
Physical Exam:
T 38.5℃ | HR 165 bpm | BP 65/40 | RR 34 bpm | SpO2 100% on room air | Wt 4.84kg
General: Flushed and warm well-nourished, well-developed infant lying in crib
HEENT: Normocephalic, atraumatic, anterior fontanelle open and flat
Neck: Supple, however, difficult to assess full range of motion due to increased stiffness and increased fussiness
Cardiovascular: Tachycardic, regular rhythm, normal S1, S2, no murmurs.
Pulmonary: Lungs clear to auscultation bilaterally, no wheezes, crackles or rhonchi. Gastrointestinal: Abdomen soft and non-tender and non-distended. Her umbilical stump was almost completely healed without erythema or discharge. She had normal bowel sounds in all 4 quadrants.
Genitourinary: Normal female external genitalia
Neurologic: She was alert, moving all extremities. The infant was fussy but consolable. Her tone was appropriate and she had normal Moro, palmar, plantar, and Babinski reflexes.
Dermatologic: No rashes or skin lesions
Lymph nodes: no significant cervical, axillary or inguinal lymphadenopathy
Diagnostics:
Complete Blood Count: WBC 20.1 with 27% neutrophils, 64% lymphocytes, 9% monocytes | Hemoglobin 15 | Platelets 399
Basic Metabolic Panel: Sodium 138 | Potassium 5.6 | Chloride 105 | CO2 23 | BUN 14 | Creatinine 0.30 | Glucose 101
Lumbar Puncture: 192,000 RBCs/mm3 | 1341 WBCs/mm3| Protein 207 mg/dL | Glucose 33 mg/dL
Chest X-ray: The cardiac silhouette is within normal limits, no focal consolidation. No vascular congestion, pleural effusion or pneumothorax.
Urinalysis: Clear yellow urine, pH: 7.0, small leukocyte esterase, negative nitrites, negative glucose, negative bilirubin, specific gravity 1.005, No RBCs or WBCs, bacteria or squamous cells
Respiratory Viral Panel: Negative for Influenza A and B, Respiratory Syncytial virus, and Covid-19
Pending studies: Urine culture, blood culture, Brain MRI, EEG

## Slide 18
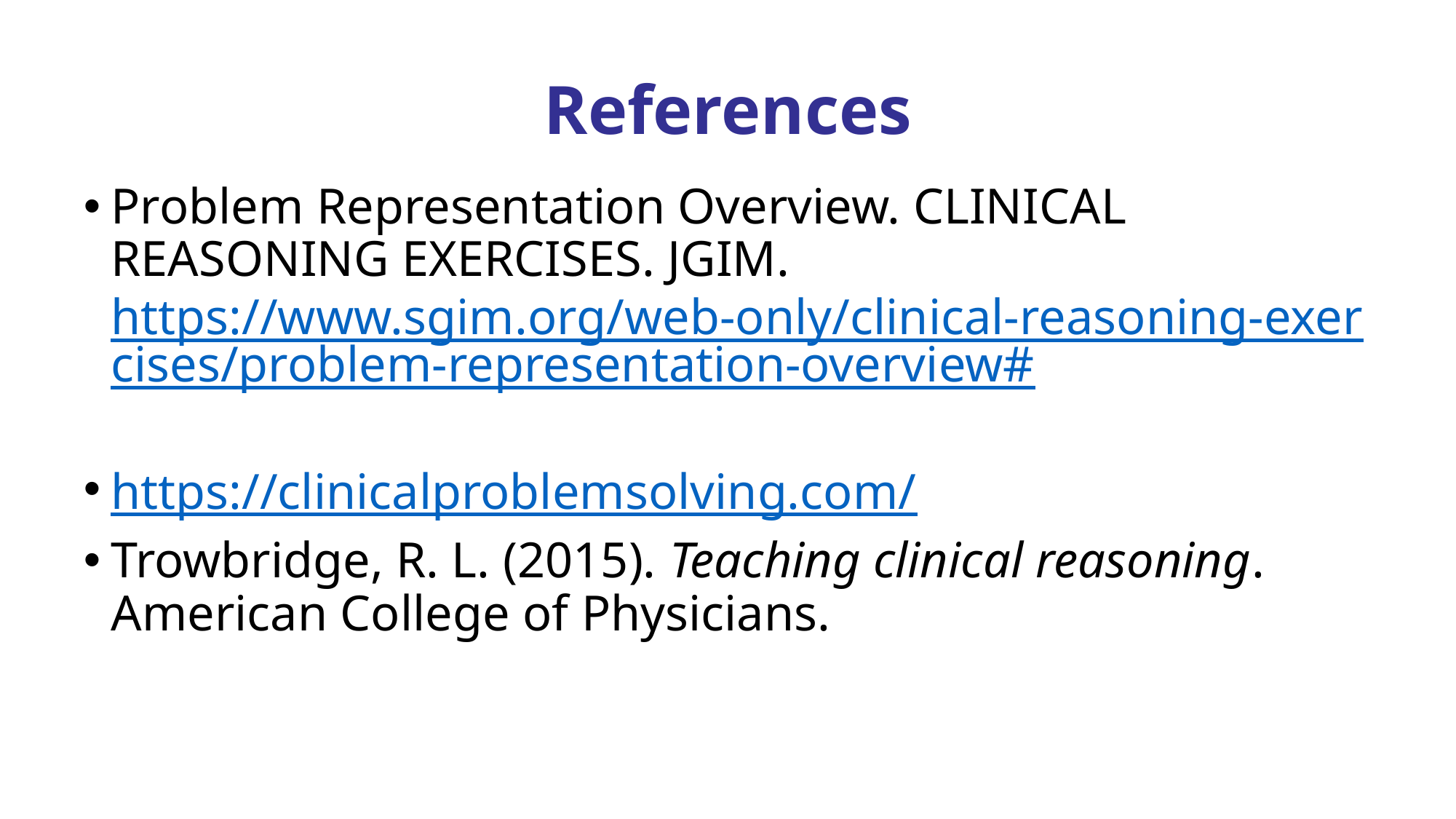

# References
Problem Representation Overview. CLINICAL REASONING EXERCISES. JGIM. https://www.sgim.org/web-only/clinical-reasoning-exercises/problem-representation-overview#
https://clinicalproblemsolving.com/
Trowbridge, R. L. (2015). Teaching clinical reasoning. American College of Physicians.

## Slide 19
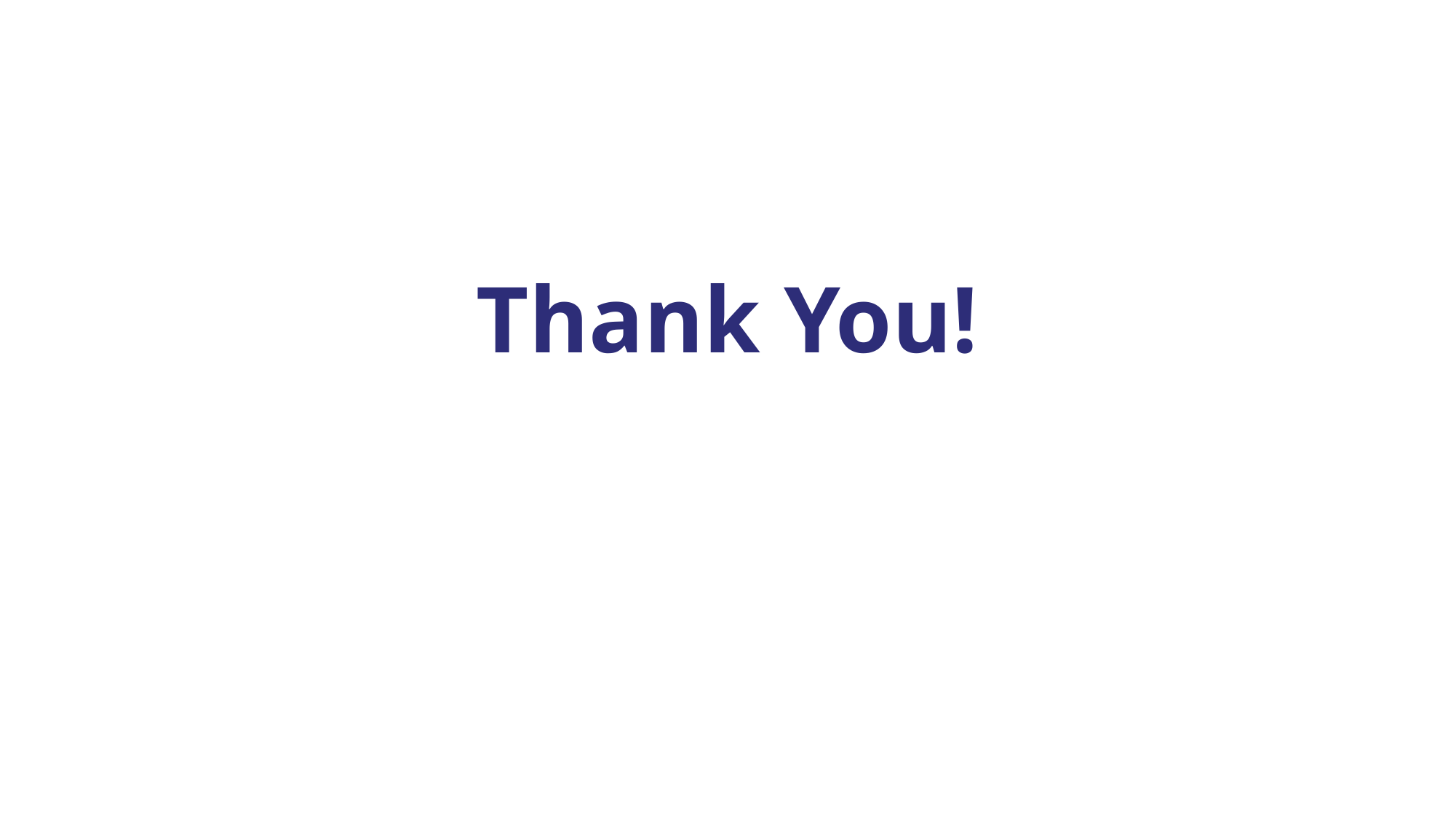

# Thank You!
