# Supplementary material for: Sum It Up for Me: A Novel Workshop in the Synthesis of Comprehensive Summary Statements for Pediatric Residents
Source: MedEdPORTAL. 2025 Nov 18;21:11555. doi: 10.15766/mep_2374-8265.11555 (PMC12623508; doi:10.15766/mep_2374-8265.11555)
Supplement: Supplementary file 1 — Participant Presurvey With Case.docxSum It Up For Me With Instructor Guide in Notes.pptxSmall-Group Cases for Instructors.docSmall-Group Cases for Learners.docParticipant Postsurvey With Case.docxSummary Statement Scoring Rubric.docx [file mep_2374-8265.11555-s001.zip › _Educational Summary Report_11555.pdf]

MedEdPORTAL® | The AAMC Journal of Teaching and Learning Resources

Original Publication

Open Access

Sum It Up for Me: A Novel Workshop in the Synthesis of Comprehensive Summary Statements for Pediatric Residents

Maritza T. Harper, MD, MS*, Muyao Lin, MA, Eliana Bonifacino, MD, MS

*Corresponding author: harpermt2@upmc.edu

Abstract

Introduction: The ACGME requires progressive monitoring of pediatric trainees' clinical reasoning. Creation of summary statements is a core clinical reasoning skill because it requires learners to prioritize patient information and express their problem representation. There is scarce formal training on the formulation of summary statements for pediatricians. We developed a workshop that provides training in developing summary statements, with the goal of increased knowledge, confidence, and skills in creating summary statements. **Methods:** The 1-hour workshop consisted of a didactic session and skills practice for creation and evaluation of summary statements. The workshop was assessed for improvement in confidence and knowledge through a pre/post workshop survey and for skills using a previously published rubric. **Results:** Twenty-four first-year pediatric trainees participated in the session. There was a 33% relative improvement in trainees' confidence in developing and assessing summary statements ($p < .001$, 95% CI, 0.0-1.0 for development; $p < .001$, 95% CI, 1.0-2.0 for assessment). There was a 26% increase in knowledge of components of a summary statement ($p < .05$). There was a statistically significant improvement in summary statement score before the workshop to immediately after: 4.40 ($SD = 1.47$) versus 5.60 ($SD = 1.34$), respectively ($p < .05$). **Discussion:** Our novel workshop demonstrated improvement in confidence in developing and assessing summary statements, improved knowledge of recommended components of a summary statement, and resulted in a statistically significant improvement in the quality of summary statements. This clinical reasoning workshop improved confidence and objectively assessed crucial clinical reasoning skills.

Keywords
Pediatrics, Summary Statements, Clinical Reasoning/Diagnostic Reasoning

Educational Objectives

By the end of this workshop, trainees will be able to:

1. Recite the core components of a summary statement, including the clinical context, a temporal pattern including the use of semantic qualifiers, and key clinical findings including use of pertinent positives and negatives.
2. Demonstrate confidence in the ability to assess summary statements to ensure that they contain the recommended components of a summary statement.
3. Generate an accurate and concise summary statement using the recommended components of a summary statement.
4. Demonstrate the effectiveness of this workshop in improving confidence, knowledge, and skill in formulating a summary statement.

Citation:
Harper MT, Lin M, Bonifacino E. Sum it up for me: a novel workshop in the synthesis of comprehensive summary statements for pediatric residents. *MedEdPORTAL*. 2025;21:11555.
https://doi.org/10.15766/mep_2374-8265.11555

Introduction

Diagnostic error, defined as an error or delay in diagnosis or a failure to act on results of monitoring or testing, has been identified as a key contributor to adverse patient outcomes.[1] Consequently, there have been national efforts to address diagnostic errors, most prominently from the National Academy of Medicine. In their landmark publication, *Improving Diagnosis in Health Care*, the National Academies of Sciences, Engineering, and Medicine identified that accurate diagnosis depends on proficiency in clinical reasoning, leading them to call for better training on the diagnostic process for all health care professionals to improve the diagnostic skills of providers, with the ultimate goal of decreasing diagnostic errors.[1,2] A physician's ability to diagnose a patient's illness is fundamental to providing correct and effective treatments. It has been described in the

Copyright © 2025 Harper et al. This is an open-access publication distributed under the terms of the Creative Commons Attribution license.

literature that an effective way to improve outcomes of the diagnostic process is to improve education for health care professionals, and that developing curricula that enhances pattern recognition, improves critical thinking, and minimizes negative effects of cognitive bias would improve real-world clinical performance.[3,4] In some studies, authors have found that educational interventions can improve diagnostic accuracy, although most have not been studied in a clinical context.[5,6] The call for clinical reasoning education on the diagnostic process has been echoed in the ACGME update for Pediatric Milestones in 2021.[7] In their recommendation, they emphasize the importance of progressive monitoring of trainees' clinical reasoning through their residency training and demonstration of proficiency in reasoning. However, limited data exist on the best methods to provide training in this domain.

In 2019, an interdisciplinary group of experts who were members of the national organization Society to Improve Diagnosis in Medicine, which represents professionals and educators from laboratory medicine, internal medicine, pediatrics, emergency medicine, nursing, pharmacy, physician assistants, medical students, and patient representatives, met to create competencies in diagnostic reasoning education for all health care professionals. Among them, one key competency identified was that of formulating an accurate problem representation expressed in a concise summary statement.[8] The ability to verbalize a problem representation through a concise summary statement is a core skill in diagnostic reasoning because it requires learners to synthesize an overall picture of the patient's presentation and prioritize patient information. Classically, the summary statement has comprised three core components: a clinical context, the temporal pattern of illness, and inclusion of key clinical findings.[9] Because the summary statement is an instrumental component of oral case presentations and clinical documentation, the ability to create a summary statement is an essential step in clinical reasoning for medical providers in all levels of training.

Although there have been numerous clinical reasoning curricula and workshops developed for medical students,[10] internal medicine trainees,[11] and emergency medicine trainees,[12] there is scarce published training for pediatricians,[13] specifically regarding guidance on the formulation of summary statements. Given that there is a clear need for teaching this important skill for pediatric trainees, we designed a novel educational workshop designed to introduce clinical reasoning concepts specifically to pediatric trainees in postgraduate year 1 and to provide opportunity for skills practice. The first year of residency may present an ideal time to receive education on this foundational skill, given that not all medical schools have a dedicated curriculum for clinical reasoning skills.[14] Providing first-year residents with an opportunity to become familiar with clinical reasoning terminology and skills practice may serve as a cognitive scaffolding[15] for further refinement as they progress through residency. The goal of this workshop was to create a session that was efficient, easy to administer, and required minimal facilitator training yet remained effective at improving knowledge, confidence, and skills.

Methods

We designed and assessed a novel workshop to provide training in the development of concise and accurate summary statements with the hypothesis that trainee participation would result in increased knowledge about, confidence in, and improved skills in the generation of summary statements.

Participants

All pediatric first-year residents were invited to participate in the workshop as a part of a regularly running didactic weekly noon conference series for interns. The University of Pittsburgh Medical Center (UPMC) Children's Hospital of Pittsburgh Pediatric Residency postgraduate year 1 class of 2023-2024 consisted of 42 trainees, including 30 categorical pediatric trainees, four internal medicine-pediatrics trainees, one pediatric anesthesia trainee, three pediatric neurology trainees, two triple Board trainees (pediatrics, psychiatry, and child and adolescent psychiatry), one pediatric scientist development trainee, and one pediatric neurodevelopmental disabilities trainee. Because this workshop was held during scheduled residency education time, and because of the various schedules of pediatric trainees, all UPMC pediatric postgraduate year 1 trainees were unable to attend the session. Although other trainees (e.g., University of Pittsburgh medical students, visiting medical students, and family medicine residents) participated in the educational session, their data were not included in the analysis.

Curricular Design and Content

We designed a 1-hour case-based workshop for pediatric residents with the goal of increasing participants' confidence, knowledge, and skills in creating summary statements and using semantic qualifiers. To demonstrate improvement in these areas through a short curricular intervention, we chose a pre-post study design. After completing a preworkshop survey (Appendix A), participants engaged in a 25-minute didactic session on the basis of the content described in the American College of Physicians' Teaching Medicine Series book, *Teaching Clinical Reasoning*[9]

Copyright © 2025 Harper et al. This is an open-access publication distributed under the terms of the Creative Commons Attribution license.

(Appendix B). This didactic session described the importance and purpose of a summary statement, outlined its components, introduced the concept of semantic qualifiers, and provided examples on how to enhance one's summary statement.

The 25-minute didactic session was followed by small group sessions in which participants reviewed pediatric cases with a facilitator, who provided explicit commentary to help participants improve and enhance summary statements for each case (Appendices C and D). For this workshop, there were a total of five facilitators. The facilitators for this session were three pediatric chief residents and two pediatric hospitalists who had undergone brief facilitator training before the session. Before the workshop, they received a 45-minute virtual facilitator training session where they reviewed the workshop materials and small group handouts (Appendices A-F). During this preworkshop training, the facilitators had the opportunity to provide feedback on the case materials and ask questions.

Each facilitator had four to six participants in each small group. The facilitator provided a copy of each case to each small group participant and allowed each small group participant to individually review the case. At the bottom of the case presentation handout, there was an example summary statement provided for the case. Using the provided facilitator guide, the facilitator guided the participants as a group to evaluate the provided summary statement for the case to ensure that it contained the recommended components of a summary statement. After completing the guided discussion, the facilitator asked one of the participants to generate a new summary statement for the case presentation, and the facilitator provided feedback to the participant. After completion of the small group session, participants completed the postworkshop survey (Appendix E). The workshop concluded with teaching points that underscored the importance of summary statement creation and specific components needed for accurate and concise summary statements.[9]

This session was designed to have a didactic session followed by relevant skills practice determined on the basis of the framework of example-based learning, defined as the process of learning or acquiring a skill by observing examples or models of that skill, which has been found to be effective for learning clinical reasoning.[16] Example-based learning is an effective component of constructivist learning theory, because it emphasizes that learners actively construct their own understanding of the world with concrete examples to analyze, which learners then use to build their knowledge and understanding.[17] Example-based learning is a widely used approach that can effectively introduce learners to new content and then provide opportunity for learners to demonstrate understanding of the principles presented.[18]

Institutional Review

This study (STUDY23070048) met criteria for exemption for educational strategies under CFR 46.104(d) by the University of Pittsburgh Institutional Review Board on July 12, 2023. Participants were informed of the study and given the opportunity to opt out of the work being used for evaluation.

Assessment

We assessed participants according to three domains: (1) confidence in creation and assessment of summary statements, (2) knowledge about expert-recommended components of summary statements, and (3) skills in generation of summary statements. A pre/post evaluation design was used to achieve these aims. Before and after the didactic content (Appendix B) and small-group session, participants were given a six-question survey (Appendices A and E) to assess their confidence and knowledge. The confidence questions were assessed using a 5-point Likert scale for each question, where 1 indicated *not confident at all* and 5 indicated *extremely confident*. The knowledge question assessed the expert-recommended components of a summary statement. The final task asked participants to review a pediatric case and write a summary statement on the basis of that case (Appendices A and E). This case was piloted with a group of pediatric chief residents before its use in the survey.

To assess the quality of summary statements written by participants, we used a previously published and validated rubric[19] developed by Smith *et al*. The rubric (Appendix F) includes five components: factual accuracy (0 = *no*, 1 = *yes*), appropriate narrowing of the differential diagnosis (0 = *does not narrow*, 1 = *some narrowing but missing elements*, 2 = *appropriately narrows*), transformation of information (0 = *none*, 1 = *some*, 2 = *frequent and appropriate*), use of semantic qualifiers (0 = *none*, 1 = *some*, 2 = *frequent and appropriate*), and a global rating (0 = *significant problems/fatal flaws*, 1 = *adequate but can be improved*, 2 = *concise, complete, accurate*). Total scores for this rubric range from 0 to 9, with greater scores indicating a greater quality summary statement. Two study authors independently evaluated the deidentified summary statements as well as whether the summary statements were pre- or postintervention was concealed from the study authors.

Data Analysis

To analyze the changes in pre- and postworkshop on confidence related to developing and assessing summary statements, we

Copyright © 2025 Harper et al. This is an open-access publication distributed under the terms of the Creative Commons Attribution license.

compared Likert scale scores using a one-tailed Wilcoxon signed-rank test. To assess the changes in participants' ability to identify the expert-recommended components of a summary statement (correct *vs*. incorrect) after the workshop compared with before the workshop, we used a one-tailed exact McNemar test to compare paired binary responses before and after the workshop.

For analyzing the difference in the total scores obtained from the rubric by Smith *et al*., the participants' skill of developing a summary statement before and immediately after the workshop, a one-tailed paired *t* test was used. The normality of quality total scoring differences between pre- and postworkshop was assessed using the Shapiro-Wilk test and visual inspection with histogram and box-plot. The percent agreement of the total scores determined by the two study authors was calculated as the proportion of summary statements for which both raters assigned an identical total score.

Analysis of the data was performed using R (version 4.3.1) for Mac OS X. Statistical significance was set at $p < .05$, with no adjustments for multiple comparisons.

Results

Participant Characteristics

After we excluded medical students and visiting non-pediatric trainees who were present at the session, 24 of 42 (57%) of eligible pediatric interns at UPMC participated in this workshop. Further stratified, the cohort of trainees that participated in the workshop included 16 of 24 (67%) of categorical pediatric interns and 8 of 24 (33%) of the combined program trainees.

Confidence

Confidence in developing summary statements and assessing summary statements, as assessed by a Likert scale question, was found to have statistically significant improvement from pre- to postworkshop. Participants' confidence in developing summary statements showed a statistically significant increase from pre- to postworkshop: median of paired difference 95% CI, 0.0-1.0 Likert level; Wilcoxon signed-rank $p = .001$. Confidence in assessing summary statements also demonstrated a statistically significant gain, median paired difference 95% CI, 1.0-2.0 Likert-scale levels; $p < .001$. Postworkshop confidence reflects an approximate 33% relative improvement from baseline, on the basis of changes in median Likert-scale levels. The pre- and postworkshop response distributions are displayed in Figure 1.

Knowledge

The expert-recommended components of a summary statement were correctly identified by 79% of participants (19/24) before and 100% of participants (24/24) after the workshop, reflecting a 26% relative increase in participant-level correctness in knowledge. A one-tailed exact McNemar test confirmed this

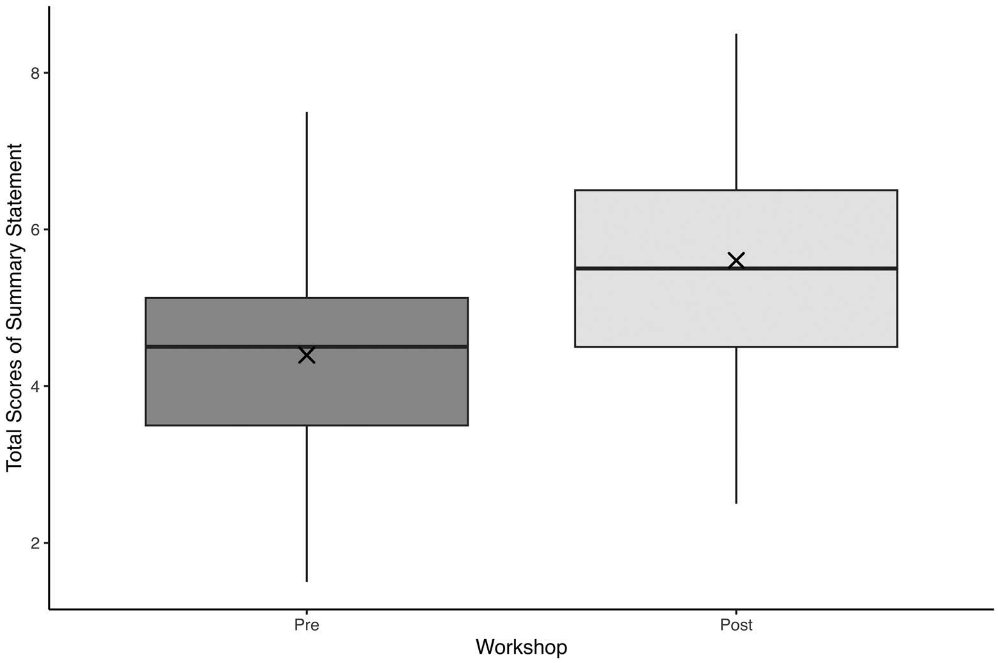

Figure 1. Boxplot of summary statement quality as measured by total score pre- and postworkshop ($N = 24$). Boxes represent the interquartile range (IQR), with horizontal lines indicating the median; whiskers denote the full range. The × marks the mean. Pre: mean 4.40 ($SD = 1.47$), median 4.50 (IQR: 3.50-5.13); Post: mean 5.60 ($SD = 1.34$), median 5.50 (IQR: 4.50-6.50).

Copyright © 2025 Harper et al. This is an open-access publication distributed under the terms of the Creative Commons Attribution license.

increase was statistically significant ($p = .03$; 95% CI, lower bound: 1.22, no upper bound), with more participants improving their knowledge of the expert-recommended components after the workshop.

Skills

When assessing pre- and immediately postworkshop summary statements quality total scoring according to the validated rubric, the Shapiro-Wilk test supported the normality of difference scores ($p = .50$). A paired t test showed a statistically significant improvement in summary statement quality ($p < .001$; paired t test, with total scores increasing from 4.40 [$SD = 1.47$] to 5.60 [$SD = 1.34$]). The percent agreement between the two raters was 53%. The total scores demonstrating the quality of the summary statements pre- and post-workshop are displayed in Figure 2.

Discussion

This study demonstrates that a curricular intervention based on the principles of example-based learning can result in improved skills in summary statement generation in addition to improved knowledge and confidence in the synthesis of summary statements. This workshop is novel, because there is currently a gap in medical literature providing a targeted session on the recommended components of summary statements to pediatric trainees.

The significant quantitative improvement in skill using the validated tool by Smith *et al.*[19] demonstrates that a short, impactful didactic intervention can improve a clinical reasoning skill. To our knowledge, this project represents one of the first effective workshops for explicitly teaching a clinical reasoning skill in pediatrics graduate medical education. Although there have been other educational-based studies in clinical reasoning,[20] none focused explicitly on summary statements, and none were specifically targeted towards residents in pediatrics. This left current educators without cases that were appropriate for pediatricians in training, leaving these educators with the option of having to pursue the time-consuming process of writing their own pediatric specific cases or using cases that were not appropriate for pediatricians. In addition, an attractive feature of this curricular intervention is that it required minimal time for facilitator training, which makes it easy for educators to integrate the session into pediatric residency programs' regularly scheduled didactics.

This study also demonstrated a statistically significant improvement in self-reported confidence of these UPMC pediatric trainees in the creation of summary statements. Notably, there was already a relatively high level of confidence before the workshop, which is consistent with previous studies of residents at the beginning of their training.[21] Although confidence and competence do not always directly correlate, our results

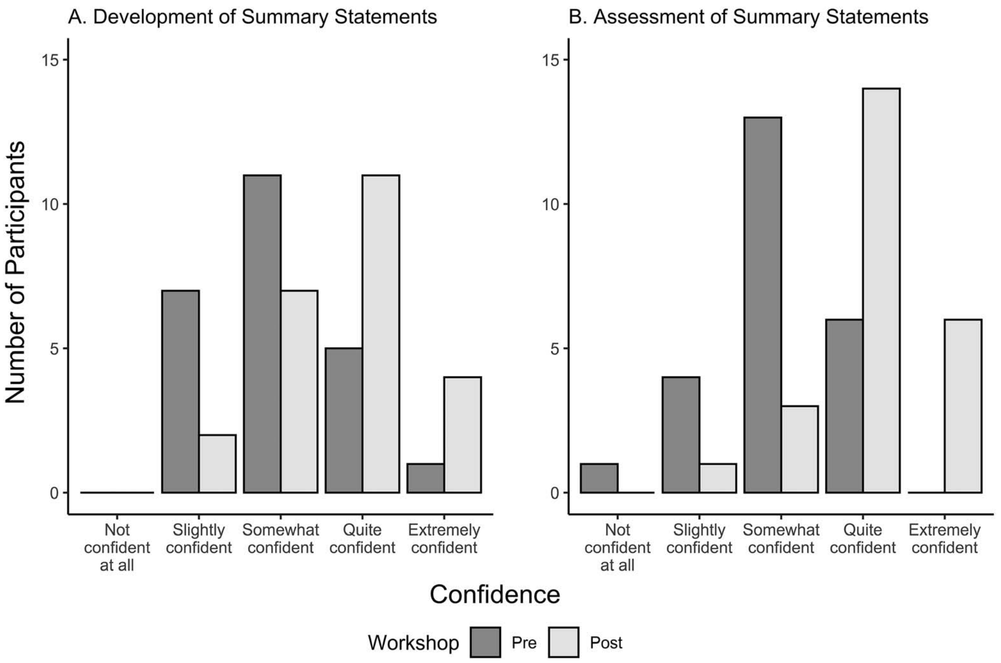

Figure 2. Bar plots of confidence levels for summary statement development (panel A) and summary statement assessment (panel B) pre- and postworkshop ($N = 24$).

Copyright © 2025 Harper et al. This is an open-access publication distributed under the terms of the Creative Commons Attribution license.

regarding the skills acquisition from this curriculum also may provide some support to improved competence with this skill.

Our workshop also improved the knowledge about recommended summary statement components in our trainees in pediatrics at UPMC. We do acknowledge that, before the workshop, trainees demonstrated some knowledge about the recommended summary statement components, which likely was attributable to knowledge obtained before their pediatrics residency.[10] Although this workshop improved the previous knowledge trainees have gained, we express caution in concluding that this improves diagnostic accuracy. For example, although the use of semantic qualifiers in experts has been associated with improved accuracy,[22] other studies have shown that simply teaching novices to use these words does not clearly improve diagnostic expertise at their level.[23] Even with this in mind, our data are a promising result because knowledge about clinical reasoning terms can help form cognitive scaffolding for further learning.[15]

Despite these promising outcomes, we recognize that this was a single-site evaluation, which limits generalizability, and also represents a one-time educational intervention; therefore, the longitudinal retention is yet to be determined. Our usage of an evaluation tool before and immediately after educational intervention has been used in many clinical reasoning studies, and we acknowledge that it would be ideal that a longitudinal assessment would be the best way to demonstrate knowledge retention of the participants. In addition, this workshop only measured outcomes using written clinical vignettes (Kirkpatrick Level 2); thus, the transfer of any effects to the real-world workplace, and ultimately to the level of patient outcomes, remains unexplored.[20] Future studies using our workshop should aim to incorporate real-world patient outcomes, but this may be difficult to study because there are few sources of valid and reliable data that enable measurement of diagnostic errors.1 Lastly, the percent agreement between evaluators of the skills assessment is only 53%, which is less than ideal, which may affect the significance of the results. Given that the rubric described by Smith *et al*. includes subjective assessments by the evaluators (narrowing of the differential diagnosis, transformation of information, use of semantic qualifiers, and global rating), this could explain the relatively low percent agreement. Next steps include developing or utilizing a more reliable and objective assessment tool for summary statements, increasing the number of evaluators of the summary statements, the creation of a longitudinal workshop that addresses other specific skills in clinical reasoning to further build on the knowledge and skills that trainees learned before residency training, as well as assessment of this curricular intervention at other sites and residency programs.

In conclusion, this interactive and novel case-based workshop is an effective means with which to teach pediatric residents about summary statements, with demonstrated evidence for improving knowledge, confidence, and skills. The success of this workshop, with its proven effectiveness at improving a skill, makes it ideal for incorporation into pediatric residency program didactics and can serve as a foundation for subsequent skills refinement in clinical reasoning. This study could contribute to the growing body of locally driven innovations leading to improvement of clinical reasoning skills among pediatric trainees.

Appendices

A. Participant Presurvey With Case.docx
B. Sum It Up For Me With Instructor Guide in Notes.pptx
C. Small-Group Cases for Instructors.doc
D. Small-Group Cases for Learners.doc
E. Participant Postsurvey With Case.docx
F. Summary Statement Scoring Rubric.docx

All appendices are peer reviewed as integral parts of the Original Publication.

Maritza T. Harper, MD, MS: Assistant Professor, Department of Internal Medicine and Pediatrics, University of Pittsburgh School of Medicine; ORCID: https://orcid.org/0009-0008-3735-3797

Muyao Lin, MA: Research Associate, Center for Biostatistics and Health Data Science, Virginia Tech

Eliana Bonifacino, MD, MS: Associate Professor, Department of Medicine, Georgetown University of School of Medicine

Disclosures
None to report.

Funding/Support
None to report.

Prior Presentations
Harper M, Bonifacino E. Sum it up for me: intern edition. A novel curriculum in the synthesis of comprehensive summary statements for pediatric residents. Presented at: Society to Improve Diagnosis in Medicine National Conference; October 2023; Cleveland, OH.

Ethical Approval
The University of Pittsburgh Institutional Review Board reviewed this project.

Copyright © 2025 Harper et al. This is an open-access publication distributed under the terms of the Creative Commons Attribution license.

Disclaimer

The views expressed in this publication are the authors' own and not an official position of the authors' institutions.

References

1. Balogh EP, Miller BT, Ball JR, eds. *Improving Diagnosis in Health Care*. National Academies Press; 2015.
2. Graber ML, Wachter RM, Cassel CK. Bringing diagnosis into the quality and safety equations. *JAMA*. 2012;308(12):1211-1212. https://doi.org/10.1001/2012.jama.11913
3. Olson APJ, Graber ML. Improving diagnosis through education. *Acad Med*. 2020;95(8):1162-1165. https://doi.org/10.1097/ACM.0000000000003172
4. Graber ML, Rencic J, Rusz D, et al. Improving diagnosis by improving education: a policy brief on education in healthcare professions. *Diagnosis (Berl)*. 2018;5(3):107-118. https://doi.org/10.1515/dx-2018-0033
5. Mamede S, van Gog T, van den Berge K, et al. Effect of availability bias and reflective reasoning on diagnostic accuracy among internal medicine residents. *JAMA*. 2010;304(11): 1198-1203. https://doi.org/10.1001/jama.2010.1276
6. Mamede S, de Carvalho-Filho MA, de Faria RMD, et al. 'Immunising' physicians against availability bias in diagnostic reasoning: a randomised controlled experiment. *BMJ Qual Saf*. 2020;29(7):550-559. https://doi.org/10.1136/bmjqs-2019-010079
7. *Pediatric Milestones*. Accreditation Council for Graduate Medical Education. Updated 2021. Accessed December 11, 2023. https://www.acgme.org/specialties/pediatrics/milestones
8. Olson A, Rencic J, Cosby K, et al. Competencies for improving diagnosis: an interprofessional framework for education and training in health care. *Diagnosis (Berl)*. 2019;6(4):335-341. https://doi.org/10.1515/dx-2018-0107
9. Ledford CH, Nixon LJ. General teaching techniques. In: Trowbridge RL, Rencic JJ, Durning SJ, eds. *Teaching Clinical Reasoning*. American College of Physicians; 2015:76-106.
10. Weinstein A, Pinto-Powell R. Introductory clinical reasoning curriculum. *MedEdPORTAL*. 2016;12:10370. https://doi.org/10.15766/mep_2374-8265.10370
11. Olenik J. *Problem Representation Overview*. Society for General Internal Medicine. Accessed December 11, 2023. https://www.sgim.org/File%20Library/JGIM/Web%20Only/Problem-Representation-Overview.pdf
12. The Clinical Problem Solvers. Accessed December 11, 2023. https://clinicalproblemsolving.com
13. Bowen J, Polak C, Thomson J, Herrmann L. A pilot longitudinal clinical reasoning curriculum for pediatric residents. *MedEdPORTAL*. 2024;20:11447. https://doi.org/10.15766/mep_2374-8265.11447
14. Rencic J, Trowbridge RL Jr, Fagan M, Szauter K, Durning S. Clinical reasoning education at us medical schools: results from a national survey of internal medicine clerkship directors. *J Gen Intern Med*. 2017;32(11):1242-1246. https://doi.org/10.1007/s11606-017-4159-y
15. Dhaliwal G, Ilgen J. Clinical reasoning: talk the talk or just walk the walk? *J Grad Med Educ*. 2016;8(2):274-276. https://doi.org/10.4300/JGME-D-16-00073.1
16. Chamberland M, Mamede S, St-Onge C, Setrakian J, Bergeron L, Schmidt H. Self-explanation in learning clinical reasoning: the added value of examples and prompts. *Med Educ*. 2015;49(2):193-202. https://doi.org/10.1111/medu.12623
17. Chand SP. Constructivism in education: exploring the contributions of Piaget, Vygotsky, and Bruner. *Int J Sci Res*. 2023;12(7):274-278. https://doi.org/10.21275/SR23630021800
18. Roelle J, Hiller S, Berthold K, Rumann S. Example-based learning: the benefits of prompting organization before providing examples. *Learn Instr*. 2017;49:1-12. https://doi.org/10.1016/j.learninstruc.2016.11.012
19. Smith S, Kogan JR, Berman NB, Dell MS, Brock DM, Robins LS. The development and preliminary validation of a rubric to assess medical students' written summary statements in virtual patient cases. *Acad Med*. 2016;91(1):94-100. https://doi.org/10.1097/ACM.0000000000000800
20. Lambe KA, O'Reilly G, Kelly BD, Curristan S. Dual-process cognitive interventions to enhance diagnostic reasoning: a systematic review. *BMJ Qual Saf*. 2016;25(10):808-820. https://doi.org/10.1136/bmjqs-2015-004417
21. Smith HL, Craig SR, Yost WJ. Examination of entering residents' self-reported confidence and supervision needs performing AAMC entrustable professional activities. *J Grad Med Educ*. 2018;10(4):474. https://doi.org/10.4300/JGME-D-18-00520.1
22. Chang RW, Bordage G, Connell KJ. The importance of early problem representation during case presentations. *Acad Med*. 1998;73(10)(suppl):S109-S111. https://doi.org/10.1097/00001888-199810000-00062
23. Nendaz MR, Bordage G. Promoting diagnostic problem representation. *Med Educ*. 2002;36(8):760-766. https://doi.org/10.1046/j.1365-2923.2002.01279.x

Received: May 8, 2025
Accepted: September 18, 2025
Published: November 18, 2025

Copyright © 2025 Harper et al. This is an open-access publication distributed under the terms of the Creative Commons Attribution license.
