# Supplementary material for: Sum It Up for Me: A Novel Workshop in the Synthesis of Comprehensive Summary Statements for Pediatric Residents
Source: MedEdPORTAL. 2025 Nov 18;21:11555. doi: 10.15766/mep_2374-8265.11555 (PMC12623508; doi:10.15766/mep_2374-8265.11555)
Supplement: Supplementary file 1 — Participant Presurvey With Case.docxSum It Up For Me With Instructor Guide in Notes.pptxSmall-Group Cases for Instructors.docSmall-Group Cases for Learners.docParticipant Postsurvey With Case.docxSummary Statement Scoring Rubric.docx [file mep_2374-8265.11555-s001.zip › D. Small-Group Cases for Learners.doc]

**CASE 1**

**History of Present Illness:**

5-year-old boy presents with 1 week of abdominal pain and frequent diarrhea. Mom denies any fevers at home. Mom is not sure if she has seen blood in diarrhea, but he has a diarrheal episode almost every 2 hours. The child describes the pain as cramping and has become increasing painful over the past 2 days. As the patient has not been able to tolerate any oral intake over the past few days, Mom brought him to the emergency department for evaluation. Otherwise, the patient has no respiratory symptoms, chills, sore throat, or complaints of ear pain.

Per mom, the patient has been coughing for 6 months, for which he is currently being evaluated by ENT. His cough acutely worsened 2 months prior to presentation without reports of sore throat. At that time, he was seen by his primary care provider, and both he and his sister (who had a sore throat) tested positive for strep. He was treated with a course of amoxicillin. After finishing the full course of amoxicillin, 2 weeks later, he endorsed sore throat and again tested positive for strep. He was treated with amoxicillin-clavulanic acid. After finishing the full course of amoxicillin-clavulanic acid, 1 week later, he returned to his primary care provider with recurrent sore throat, and was treated with a course of cefdinir after a positive strep test. One week prior to admission, he saw his primary care provider for follow up of recurrent strep pharyngitis when he denied throat pain, but reported some neck pain. He was again positive for strep, and was treated with a course of clindamycin which he completed the day prior to admission.

Separately, he was also diagnosed with croup 2 weeks prior to admission and finished a 3-day course of prednisolone.

Review of symptoms were negative except as noted above.

**Past Medical History:** GERD, recurrent acute otitis media (AOM), submucosal cleft, acquired genu valgum of both knees, language delay

**Past Surgical History:** Bilateral myringotomy tubes, frenectomy, tongue papilloma resection

**Birth History:** He was born full term without complications; he did not require a NICU stay

**Developmental History:** He has been receiving physical therapy/occupational therapy/speech therapy for language delay, feeding difficulties, and genu valgum; has bilateral ankle braces and shoe inserts

**Family History:** Non-contributory

**Allergies:** NDKA, walnuts, environmental allergies

**Medications:** famotidine 2.8mg by mouth BID

**Social History:** The child lives at home with his mother, father, and older sister.

**Immunization History:** Up to date on age-appropriate vaccines, not vaccinated against COVID

**Physical Exam:**

**Temperature** 36.8℃ | **Heart Rate** 125 beats per minute | **Blood Pressure** 94/69 | **Respiratory Rate** 24 breaths per minute |

**SpO2** 100% on room air | **Weight** 17.9kg

**General:** No apparent distress, well developed, well nourished.

**HEENT:** Normocephalic, atraumatic, left ear tube in place, right tympanic membrane with serous fluid layering without erythema, right ear tube not visualized. Moist mucous membranes.

**Neck:** Neck soft and supple, no masses or cervical or supraclavicular lymphadenopathy.

**Cardiovascular:** Regular rate and rhythm, normal S1, S2, no murmurs, rubs or gallops. Capillary refill less than 2 seconds. 2+ dorsalis pedis pulses bilaterally, extremities warm and well perfused.

**Pulmonary:** Normal work of breathing, clear to auscultation bilaterally, no wheezes, rales or rhonchi, good air movement bilaterally.

**Gastrointestinal:** Abdomen soft, generalized tenderness in all 4 quadrants, no rebound tenderness. Hyperactive bowel sounds. Abdomen non distended. No hepatosplenomegaly.

**Rectal exam:** Bright red blood mixed with stool

**Genitourinary:** Deferred.

**Neurologic:** Alert, moving all extremities spontaneously. Normal tone and strength, no focal deficits noted.

**Dermatologic:** Warm, normal color and turgor. No rashes, lesions or wounds.

**Diagnostics:**

**Basic Metabolic Panel:** Sodium 138 | Potassium 3.7 | Chloride 105 | CO_2_ 26 | BUN 14 | Creatinine 0.33 | Glucose 72

**Complete Blood Count:** White Blood Cell 15.2 | Hemoglobin 9.4 | Hematocrit 19.2 | Platelets 310 | Neutrophil % 79

**Summary Statement:**

5-year-old boy with past medical history of GERD, recurrent AOMs, persistent cough and multiple recent courses of antibiotics for persistent strep pharyngitis who presents with 1 week of abdominal pain and diarrhea.

**CASE 2**

**History of Present Illness:**

9-month-old male who presents with increased work of breathing, wheezing and cough. Mom reports that 5 days prior to presentation, he developed wheezing that consistently occurred almost every hour. It improved with albuterol and mom began to use his albuterol inhaler, 2-3 puffs every 3 hours which worked well. However, 2 days prior to admission, he developed a non-productive cough and increased work of breathing. During this cough, he would turn extra red in the face and seemingly “choke” on his saliva. Otherwise, he has not had any fevers or issues with his feeds. Due to continued coughing and concern for choking, mom brought him in to the emergency department for evaluation. Mom reports that his 3-year-old brother, who goes to daycare, also started wheezing and coughing at the same time, but has been doing well with his own albuterol treatments.

Mom says he has otherwise been doing well, he recently switching formula two weeks ago from a premature infant 22kcal to a term infant 20kcal formula due to increasing weight velocity. He is tolerating 150mL run over 90 minutes via G-tube.

1 month prior, the infant was admitted to the hospital for increased work of breathing in the setting of rhinovirus/enterovirus infection; he was treated with supplemental oxygen via nasal cannula, albuterol, ipratropium bromide, and IV methylprednisolone, and was discharged home with an albuterol inhaler.

Review of symptoms were negative except as noted above.

**Past Medical History:** History of bilateral subdural hematomas, epilepsy with history of status epilepticus resulting in hypoxic ischemic encephalopathy (HIE), slow weight gain, dysphasia with silent aspiration, history of provoked left iliofemoral venous thrombosis

**Past Surgical History:** G-tube placed at 4 months of life, left inguinal hernia repair

**Birth History:** Former 28-week male, required bubble CPAP until 62 days of life (never required intubation). He remained int the NICU for 10 weeks and was discharged on room air.

**Developmental History:** Global developmental delay, unable to sit up independently, is not able to cruise, truncal hypotonia.

**Family History:** Older brother, mother and maternal grandmother with asthma

**Allergies:** NDKA

**Medications:** Levetiracetam 300mg BID, oxcarbazepine 120mg BID, gabapentin 30mg BID, famotidine 3mg BID, senna 2.2mg daily, cholecalciferol 400 units daily, liquid multivitamin 1mL daily, albuterol 2 puffs every 4 hours as needed for wheezing; all non-inhaler medications are administered via G-tube.

**Social History:** The child lives at home with her mother, father, and 3-year-old brother

**Immunization History:** Up to date on age-appropriate vaccines, he has not received the seasonal COVID vaccine

**Physical Exam:**

**Temperature** 37.2℃ | **Heart Rate** 128 beats per minute | **Blood Pressure** 115/69 | **Respiratory Rate** 70 breaths per minute | **SpO2** 98% on room air | **Weight** 9.13kg

**General:** Well-developed infant snoring while sleeping in bed in mild respiratory distress

**HEENT:** Normocephalic, atraumatic, moist mucous membranes, nasal congestion present

**Cardiovascular:** Regular rate and rhythm, normal S1, S2, no murmurs, rubs or gallops. Capillary refill less than 2 seconds. 2+ pulses in all four extremities, warm and well perfused.

**Pulmonary:** Prolonged expiratory wheezing in all lung fields, increased work of breathing noted, coarse breath sounds throughout. Occasional subcostal retractions but no head bobbing. No rales appreciated.

**Gastrointestinal:** Abdomen soft, non-tender and non-distended. No hepatosplenomegaly. G-tube site non-erythematous

**Genitourinary:** Contact dermatitis and erythema noted in inguinal skin folds and gluteal folds

**Neurologic:** Alert, moving all extremities spontaneously. Truncal hypotonia

**Diagnostics:**

**Respiratory Viral Panel:** Positive for respiratory syncytial virus (RSV)

**Summary Statement:**

9-month-old with history of NICU stay requiring bubble CPAP, history of chronic bilateral subdural hematomas, history of status epilepticus and subsequent hypoxic ischemic encephalopathy now well controlled on seizure medications, slow weight gain s/p G-tube placement, and recent hospitalization for rhinovirus/enterovirus bronchiolitis with reactive airway disease element who presents with wheezing.

**CASE 3**

**History of Present Illness:**

5-year-old male presents with 1 day of fever, vomiting, and diarrhea. 1 day prior to admission, he developed vomiting and diarrhea. He has had approximately 4-5 episodes of non-bilious, non-bloody emesis and 4 episodes of non-bloody diarrhea. He had 1 episode of fecal incontinence; mom says he recognized the urge to have a bowel movement but said he was too weak to get to the bathroom in time. He has also been febrile since yesterday morning with a max temperature of 103.8℉ at home. Mom attempted to give acetaminophen, but the patient vomited shortly after medication administration. His oral intake has been significantly decreased - mom says he has only eaten a few bites of solid food in the past 24 hours and has been able to tolerate very minimal fluid intake. Last void was the evening prior to admission. Mom says that earlier this week, the patient’s sister was ill with vomiting and diarrhea, but her symptoms resolved within 3-4 days.

Mom also reports rhinorrhea, nasal congestion, and that patient has been snoring excessively even when well, has complained of sore throat for weeks. Review of symptoms were negative except as noted above.

**Past Medical History:** 3-methylcrotonyl-CoA carboxylase (3-MCC) deficiency diagnosed via state newborn screen, followed by Medical Genetics, obesity (BMI > 99^th^ %ile), mild tricuspid regurgitation, followed by Cardiology

**Past Surgical History:** None

**Birth History:** Former 39-week male born via vaginal delivery, NICU stay for 2 days due to temperature instability.

**Developmental History:** Met all developmental milestones on time, he is very physically active

**Family History:** Older sister with 3-MCC

**Allergies:** NDKA

**Medications:** Levocarnitine 800mg BID

**Social History:** The child lives at home with his mother, father, and 3 older siblings. He does not attend school or daycare.

**Immunization History:** Not up to date; received 1 dose of Hepatitis B vaccine, Diphtheria, Tetanus, Pertussis vaccine, and Polio Vaccine in November 2018

**Physical Exam:**

**Temperature** 38.6℃ | **Heart Rate** 160 beats per minute | **Blood Pressure** 72/50 | **Respiratory Rate** 28 breaths per minute | **SpO2** 97% on room air | **Weight** 35kg

**General:** Ill-appearing 5-year-old male lying in bed with arms and legs outstretched. Responds to questions appropriately with head nods or 1–2-word answers.

**HEENT:** Normocephalic, Atraumatic. External ears normal bilaterally. Tympanic membranes dull bilaterally (right > left) but not bulging. PEERL. EOMI. dry mucus membranes, nasal congestion, dried yellow/green nasal secretions on the right cheek. Full range of motion of the neck and no tenderness to palpation.

**Cardiovascular:** Tachycardic, regular rhythm. II/VI systolic murmur heard best at the upper sternal border**.** Cap refill >4. 2+ pulses palpable in extremities and groin, extremities are cool to touch.

**Pulmonary:** Poor participation with exam, aeration poor, no wheezes or crackles noted. No retractions appreciated. Referred upper airway sounds appreciated.

**Gastrointestinal:** Protuberant abdomen, soft and non-tender and non-distended. No hepatosplenomegaly.

**Genitourinary:** Normal external male genitalia, uncircumcised

**Neurologic:** Somnolent, responds to voice and pain, will not cooperate for neuro exam, will move extremities spontaneously, but when asked to perform tasks, says he is too weak.

**Diagnostics:**

**Basic Metabolic Panel:** Sodium 136 | Potassium 6.1 | Chloride 110 | CO_2_ 20 | BUN 12 | Creatinine 0.62 (baseline 0.3) | Glucose 118 |

Magnesium 2.0

**Complete Blood Count:** White Blood Cell 29.9 | Hemoglobin 13.2 | Hematocrit 39.7 | Platelets 310 | Neutrophil % 79

**Procalcitonin:** 0.26

**CRP:** 6.31

**Respiratory Viral Panel:** Negative

**Summary Statement:**

5-year-old male with 3-methylcrotonyl-CoA carboxylase deficiency presenting with 24 hours of fever, vomiting, and diarrhea with exam notable for elevated heart rate, weakness and somnolence and labs notable for marked neutrophil predominant leukocytosis.
